# Cryopreservation of human lung tissue for 3D ex vivo analysis

**DOI:** 10.1186/s12931-025-03265-y

**Published:** 2025-05-15

**Authors:** Nickolas G. Diodati, Ganlin Qu, Borna Mehrad, Matthew A. Schaller

**Affiliations:** Division of Pulmonary, Critical Care, and Sleep Medicine, University of Florida College of Medicine, 1200 Newell Drive, Room MSB-M440, Gainesville, FL 32610 USA

**Keywords:** Cryopreservation, 3D culture, Ex vivo, Tissue culture, Human lung culture

## Abstract

Ex vivo culture techniques have assisted researchers in narrowing the translational gap between the lab and the clinic by allowing the study of biology in human tissues. In pulmonary biology, however, the availability of such tissues is a limiting factor in experimental design and constrains the reproducibility and replicability of these models as scientifically rigorous complements to in vitro or in vivo methods. Cryopreservation of human lung tissue is a strategy to address these limitations by generating cryopreserved biobanks of donors in the ex vivo study of pulmonary biology. Modern cryopreservation solutions, incorporating blends of cryoprotective extracellular macromolecules and cell-permeant non-toxic small molecules, have enabled the long-term storage of human lung tissue, allowing repeated experiments in the same donors and the simultaneous study of the same hypothesis across multiple donors, therefore granting the qualities of reproducibility and replicability to ex vivo systems. Specific considerations are required to properly maintain fundamental aspects of tissue structure, properties, and function throughout the cryopreservation process. The examples of existing cryopreservation systems successfully employed to amass cryobanks, and ex vivo culture techniques compatible with cryopreservation, are discussed herein, with the goal of indicating the potential of cryopreservation in ex vivo human lung tissue culture and highlighting opportunities for cryopreservation to expand the utility of ex vivo human lung culture systems in the pursuit of clinically relevant discoveries.

## Introduction: ex vivo lung models in pursuit of improved translational capacity

For as long as pulmonary biology has been studied, researchers have attempted to determine the mechanistic causes of lung diseases by replicating characteristics of the human lung in a laboratory environment. This approach has primarily involved the utilization of genetic manipulation or disease models derived from the culture of cells or animals [1–4]. While these models have advanced contemporary biology by providing a wealth of mechanistic information on pulmonary disease at a molecular level [5–7], the translation of these findings into clinical efficacy has demonstrated mixed success [8–10]. With around 80% of drugs found to be effective in murine studies ultimately failing clinical trials in humans [11, 12], and a success rate of between 50 and 60% in clinical trials of lung cancer therapies originating from animal studies [13], there is a significant need for additional models to improve the correlation between laboratory observations and clinical outcomes [10, 11, 14]. Ideally, new models will improve upon in vitro techniques by including features such as a retention or recreation of the lung’s characteristic 3D structure [15–17], varied cellular composition [18, 19], interactions between the more than 60 constituent lung cell types [20–22], relevant mechanical forces and stimuli [20, 23, 24], response to perturbation or infection [25, 26], and elements of any chosen disease states [27, 28]. Reproducing these attributes with as much fidelity as possible to a functional human lung is critical in ensuring in vitro results will properly translate to clinical applications [29, 30]. Recent advances have seen the development of ex vivo human lung culture techniques emerge as a method for satisfying these criteria [25, 31–33].

With the appropriate range of human lung cells arranged in a natural 3D architecture [31], ex vivo culture techniques can be utilized to study traits of human pulmonary biology [34, 35], response to drugs [36–38] or pathogens specific to humans [18, 39], or investigate the early stages of diseases which are either difficult to faithfully model in animals [40, 41] or challenging to observe in humans [42, 43]. Choice of ex vivo culture method will depend on factors such as availability of tissue, resources, and research question [44]. Common ex vivo lung culture models include human precision-cut lung slices (hPCLS) [45], culture of explanted lung tissue fragments [32], decellularized lung tissue [34], and growth of organoids derived from primary lung cells [29]. Each of these methods has strengths which suit them to different aspects of pulmonary biology, but all have the advantage of utilizing primary human cells in three dimensions for the study of lung disease [44].

In addition to the wealth of potential methods available for ex vivo culture of the human lung, there are numerous reasons ex vivo culture is increasingly employed in the study of human pulmonary biology [4, 25, 44]. Generally, utilization of human specimens is advantageous when the study requires a fidelity to in vivo human lung biology difficult to accurately replicate in animals, such as in viruses specific to human hosts [18, 39, 46, 47]. In this scenario, ex vivo explant culture provides a method for directly observing host-pathogen interactions in the human lung’s component cells [46, 47] within a 3D model containing a natural extracellular matrix (ECM) and resident immune cells [18, 48], a configuration not reproducible in two-dimensional cell culture [49]. With this 3D nature also comes the potential to mimic the breathing motions found within an active lung [24, 50, 51], responsible for critical mechanical stimuli which govern the balance of Type and Type II alveolar epithelial cell (AECI and AECII) identity [52] and extracellular matrix composition [53] in these cellular relationships, and essential to the proper modeling of inhaled drug delivery [54]. In addition, there are various physiological differences between animal models of the lung and human lung models [10, 55], not all of which are known [56–58]. In the case of diseases such as cancer, ex vivo culture of organoids or explants can also incorporate autologous immune cells, which is impossible in cell lines [59], recapitulate spatially dependent features of tumorigenesis [60], or function as a personalized testbed for immunotherapies [61]. For these reasons, ex vivo culture methods are often suggested as a way to bridge the translational gap between the lab and the clinic, improving correlation between lab studies and the outcomes of clinical trials [62].

Despite the abundance of potential for ex vivo lung culture to produce new models with improved clinical correlation, there are some limitations to these methods. The numerous physical [4, 45, 63], chemical [64], and biological [65, 66] discrepancies which naturally arise during the culture of human lung cells and tissue fragments outside of their native milieu present challenges in the correct modeling of in vivo outcomes and mechanisms [44]. By necessity, all of these models will also only partially represent a region of the lung rather than the entire tissue or organ, limiting conclusions derived from experiments involving them to local effects [66]. Several ex vivo models do not incorporate mechanisms for waste metabolite clearance or circulation, the forces of which provide critical signaling cues in biological processes such as coagulation [67, 68]. This lack of circulation precludes ex vivo models from modeling systemic effects, such as toxicity from a drug treatment stemming from reactivity in other organs, or whole-body immune system dynamics relating to immune cell migration in the context of infection, as the majority of ex vivo models only represent a single organ [45]. Some of these models, such as organoids, are also highly sensitive to the composition of culture media [69, 70]. The density of tissue cultured ex vivo and the necessity of culture media also places many ex vivo lung tissue models in what is at best a partially hypoxic environment, potentially creating challenges for the analysis of certain hypotheses [71]. The formation of specific hypotheses, adapted to the limitations of each model, is thus necessary to ensure relevance and correct translation to clinical outcomes [72].

Ex vivo tissue culture is impossible without access to high-quality donor tissue for experiments, especially those which require multiple replicates [66]. Obtaining human lung tissue requires coordination between clinics and potential donors, and is often time-consuming and laborious [73]. Throughout the entire tissue collection process, care must be taken to document variables which could potentially affect the characteristics of the recovered sample and subsequent experimental results, necessarily including but not limited to patient health history and diagnoses associated with the collected tissue, location and condition of the collected tissue from the donor’s lungs, media formulation and temperature used to transport the tissue, warm ischemia time prior to sample collection, method and instrument of tissue dissection, amount of elapsed time prior to dissection, dimensions and thickness of dissected tissue, length of adaptation period, and tissue viability prior to and following any storage [74]. In addition, cell and tissue composition of the lung is functionally distinct across the alveoli, bronchioles, and bronchi, so the lobe of origin and presence of airways or amount of parenchyma in tissue collected and prepared for ex vivo culture should be considered [22, 74, 75]. As the majority of available lung donors are likely to belong to a disease population, the effects of disease history are also a potential variable in the use of their tissue [66], with historical factors such as smoking or drug treatment history particularly noted for their effects on the tissue received [76, 77]. In certain cases, the availability of diseased tissue may be desirable, such as in the usage of lung recovered from patients with idiopathic pulmonary fibrosis [78, 79] or COPD [80]. Apart from these elements, one of the primary limitations of ex vivo lung models is tissue scarcity, which constrains the ability of these models to offer the scientifically rigorous elements of reproducibility (the ability to obtain the results of multiple experiments within the same donor) and replicability (the ability to obtain the results of the same experiment in multiple donors) [81].

## Cryopreservation to enhance the utility of ex vivo lung models

Cryopreservation has the potential to address the scarcity of high-quality human lung donor tissue for ex vivo models and improve their inherent reproducibility and replicability weaknesses [82–84]. In addition to storing tissue available at the researcher’s convenience [85], cryobanks allow for the study of multiple donors in parallel [84], the opportunity to observe effects potentially related to donor heterogeneity [18], and afford a platform of study suited to high-throughput experiments [82]. Perhaps most advantageous compared to the use of fresh tissue, cryobanks provide the benefit of allowing repeat experiments to be performed on the same donor, which would otherwise be impossible [18]. These advantages have prompted researchers to amass cryobanks as a basis for clinically relevant studies [76]. However, just as tissue culture in three dimensions presents numerous challenges which do not apply to two-dimensional cell culture [31], there are unique considerations for which researchers must account in the cryopreservation of 3D tissues and organoids. To appropriately address these considerations, a thorough understanding of the principles of cryopreservation [86] is necessary.

For successful cryopreservation, cells must be protected from injury caused by the numerous phase transitions implicit in transfer to and from a sub-freezing environment [87]. The loss of cell viability at or below sub-freezing temperatures during cryostorage occurs due to the mechanical disruption induced by ice crystal formation [88, 89] inside and outside of cells [90, 91], and most cryoprotectants function by preventing the destructive effects of ice crystal formation on critical cellular structures such as membranes [92, 93]. Additional damage to freezing or thawing cells is caused by the cellular sequestration of salt during the formation of extracellular ice crystals, effectively dehydrating cells and inducing severe volume reduction [94, 95] prior to intracellular aqueous salt crystallization and further deleterious effects [96]. The severity of these crystallization events is generally dependent on the rate of cooling [97] throughout the cryopreservation process, as a solution of cooling or heating cells passes through temperature points at which phase changes occur and crystallization or recrystallization is induced [92, 98]. The optimal rate of cooling depends on the type of cryoprotectant used [87]. Slow cooling occurs at a rate of -1 °C/min in specialized containers, whereas vitrification, or fast-cooling, occurs at rates exceeding − 20,000 °C/min [99]. Cooling rate must be considered in the context of osmotic stresses placed upon cells by the permeating components of a cryoprotectant [100], and effective cryoprotectants will ease the mechanical stresses induced by these events over all the phase changes during freezing and thawing in such a way that the plasma membrane remains intact and the overall viability of the cryopreserved cells is not compromised [88, 92, 101].

The earliest discovered cryopreservation methods involved the use of cell-permeant small molecules which prevent cell lysis or damage during freeze-thaw cycles, such as glycerol [102] or DMSO [103]. Despite the widespread, nearly century-long success of these compounds at preserving a wide variety of cells at sub-freezing temperatures [102], these cryopreservatives are poorly suited to some applications [97]. DMSO, for instance, poorly maintains functional macrophages through a freeze-thaw cycle, heavily impacting their ability to generate reactive oxygen species [104], and in some cases inhibits the differentiation potential of embryonic stem cells [105]. Furthermore, there are numerous reports of broad cytotoxic [106], transcriptional [107], and epigenetic [108] side-effects associated with the use of DMSO in various cell types [109]. Glycerol is less toxic than DMSO overall [110], but its high viscosity poses handling challenges upon thawing [111] and alters intracellular protein interactions [112]. In cryopreservation of tissues, which are by nature comprised of a heterogeneous cellular constituency, the differential effect of cryoprotectants on viability and function also varies depending on cell type, with neutrophils and primary fibroblasts notably affected by the cryopreservation process, especially when DMSO is used [113, 114]. Researchers should therefore accordingly account for the uneven alteration of cellular viability and function when assessing the suitability of a cryopreserved ex vivo model to meet research objectives [74]. The detrimental effects observed in cryoprotectants such as DMSO and glycerol have prompted exploration of alternate cryopreservatives for use in research applications where these drawbacks would prevent meaningful observations, potentially with a more consistent preservation of all cell types present in tissues [93, 97].

A major area of focus in the development of new cryopreservatives is that of biologically compatible macromolecules, inspired by their utilization in extremophiles to withstand inhospitably cool temperatures [93, 97, 115, 116]. Nature has served as a guide for the study of several of these macromolecular cryoprotectants [117], with polyols and sugar polymers some of the first observed to help larger organisms such as insects and amphibians survive freezing temperatures [118–120]. Types of macromolecular cryoprotectants which have seen laboratory success in preserving viable cells at rates similar to that of DMSO or glycerol include polysaccharides of trehalose [116, 121, 122], sucrose [116], and inulin [123], polymers which contain mixed charges to assist in maintenance of cellular osmotic integrity (polyampholytes) [97, 124] such as proteins [125], or other polymers such as polyethylene glycol [126], polyvinyl alcohol [127], or polyvinylpyrrolidone [128]. In addition to these biologically derived compounds, hydrogels of agarose [129, 130], gelatin, and alginate [131], materials used in the preparation of PCLSs [132], have also been successfully employed as cryopreservation agents [133]. Whereas small-molecule cryoprotectants act by restricting ice crystal formation and regulating osmotic pressure inside and outside of cells [98, 100, 134], macromolecular cryopreservatives are not cell-permeant and function by controlling the flow of solutes and water into and out of cells during the freezing process, effectively preventing excess dehydration associated with extracellular ice formation while still allowing enough dehydration to prevent the formation of intracellular ice crystals and maintenance of cell size [124]. Macromolecules containing mixed charges have also been presumed to act in a manner which preserves the integrity of the cell membrane or other intracellular structures, such as proteins or microtubules, independent of any ability to restrict ice crystal formation during the freezing and thawing process [97, 135, 136]. Within these molecules, charge ratios and the location of charges are critical to their ability to maintain viability of cryopreserved cells [136, 137]. Hydrogel-based cryoprotectants such as those of agarose function by reducing free water and mechanically inhibiting ice crystal formation during freezing while also restricting recrystallization of ice during thawing [129].

Despite the effectiveness of some biologically derived macromolecular cryoprotectants in their host species, these compounds do not achieve biological efficacy in isolation [97], and have been shown to inadequately protect the cell membrane from the formation of sharp extracellular ice crystals when used alone as cryoprotectants [138]. This would indicate that any osmotic control or membrane alteration provided by these compounds is not alone enough to safeguard cellular viability during the freezing and thawing process, and biological mechanisms of tissue cryoprotection rely on the presence of both large and small molecules [139]. Indeed, this phenomenon can be observed in frogs, where a blend of polysaccharides and urea balances the osmotic gradient of solutes within cells and prevents cell shrinkage during freezing due to the departure of water, enabling survival in harsh freezing temperatures [140]. Examples such as these, which combine cryoprotective macromolecules and small molecules, serve as a guide for how cryoprotectants utilized in a laboratory setting could be designed to improve control over detrimental osmotic effects while mitigating intracellular ice formation and cell dehydration [141]. The properties of these biological examples have become a template in the design of synthetic cryoprotectants, such as zwitterionic small molecules [134], which could pair with emerging synthetic macromolecular compounds to achieve the goal of finding non-toxic and biologically inert cryopreservation solutions [87].

Small-molecule and macromolecule cryoprotectants, as well as solutions containing combinations of both, have been shown to effectively cryopreserve ex vivo lung tissue for on-demand culture [18, 77]. As air comprises 80% of lung tissue, most conventional small-molecule cryopreservation solutions such as DMSO easily diffuse through the tissue mass and displace harmful ice crystal formation, enabling a consistent freezing point propagation throughout the explanted tissue that is within 10% of that found in other solid tissues [142]. The choice of appropriate cryopreservation technique and methods with which to validate effectiveness of cryopreservation will vary, however, depending on which ex vivo lung model is employed [77]. While cell-permeant cryopreservatives like DMSO are an appropriate media in which to preserve hPCLSs, for example, these same cryopreservatives may be incapable of penetrating denser explants at a high enough concentration for desired levels of viability after exposure to temperatures found in the vapor phase of liquid nitrogen [18, 143]. Below, ex vivo lung culture techniques suitable for cryopreservation and cryobank accumulation are discussed, as well as the appropriate cryopreservation techniques for each and notable descriptive or hypothesis-driven studies which have been performed using viable lung tissue from cryobanks.

## Ex vivo lung models which can be cryopreserved

### Human precision-cut lung slices (hPCLSs)

**Fig. 1:**
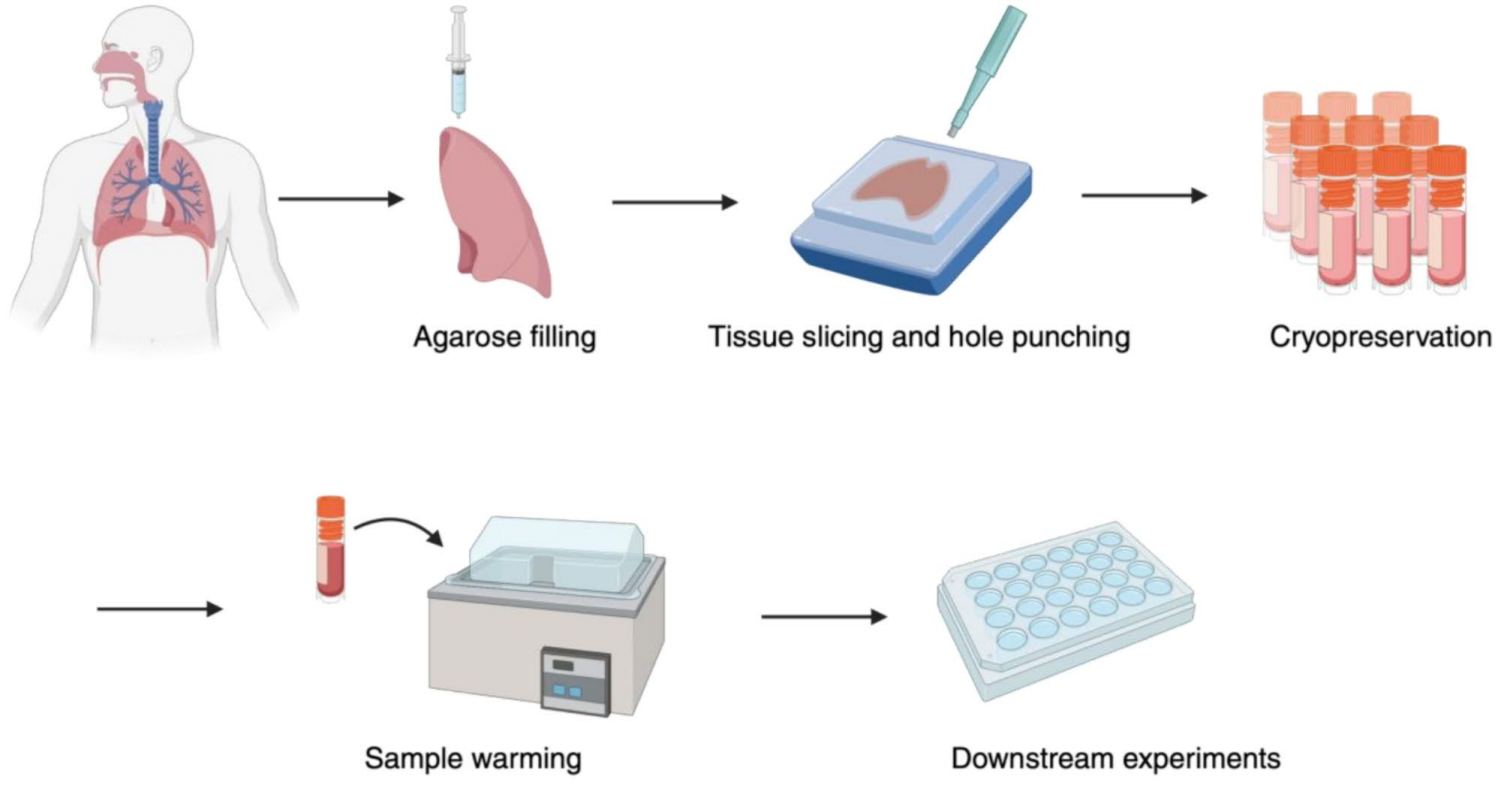
Diagram illustrating the cryopreservation of human precision-cut lung slices for cryobank generation and experimental use. The thin nature of precision-cut lung slices enables the mass storage of several slices from the same donor in a multitude of cryoprotectants. Figure illustrations were generated in BioRender

Perhaps the most-studied model in cryopreservation of ex vivo human lung tissue is that of precision-cut lung slices, sections of lung tissue measuring around 1 mm or less in thickness [144]. PCLSs were first employed in animal studies of lung toxicology during the 1980s [145, 146] before being utilized in studies of human toxicology in 1994 [147]. hPCLSs are generally best obtained from a fresh lobe of lung which has been inflated with low melting point agarose or gelatin to prevent the collapse of the alveoli, sectioned, and hole-punched, before the hole punches are sliced by a vibratome or other precision slicing instrument to produce slices of the desired thickness [45]. Several slices can be created from each hole punch, and several hole punches can be taken from each section, generating a large number of replicates from a given region of the lung in the same patient [45, 63]. In addition to the parameters which should be considered in the collection of any tissue for ex vivo culture, characteristics of collected hPCLSs, including composition of filling gel, gel temperature, tissue sampling method, dimensions of tissue selected for slicing, elapsed time prior to slicing, resultant PCLS thickness, and slicing instrument, blade, and angle should be documented to account for potential variability [74]. Each hPCLS represents a near-3D spatial snapshot of the donor lung, complete with any airways, blood vessels, or parenchyma present in the hole punch [148], a full complement of cellular heterogeneity including structurally critical cells and resident immune cells [149, 150], an air-liquid interface [151], the potential inclusion of mechanical stimuli associated with breathing [24, 50, 51], and the ability to function as a model of physiological phenomena such as airway constriction [152–156]. In addition to their usefulness in toxicology and drug discovery studies [74, 157], these features have made hPCLSs an excellent model for the study of a wide variety of lung diseases, including bacterial [25] and viral [149] infections, chronic inflammatory disorders [4], allergic reactions [158], and cancer [159]. hPCLSs can also serve as a platform for comparing murine and human response to the same disease [160], and can be generated from healthy or diseased donors, meaning that disease phenotypes can either be studied directly as they existed in situ or induced experimentally for analysis [28, 161].

Despite the utility of the hPCLS model, there are limitations which must be considered in the course of experimental design [45]. Though the thinness of hPCLSs allows for the formation of an air-liquid interface, the 3D structural detail of this model is limited compared to that of tissue fragments, potentially making that model a better choice for studies which require greater spatial detail [18, 144]. This lack of dimensionality in hPCLSs also allows any factors with which they are treated in culture media, including viruses or drugs, to pass above epithelial barriers and reach all cells of the slice in a way that would not occur physiologically [45]. While it was initially believed that culture of PCLS was limited to less than 7 days without specific culture conditions, such as within hydrogels coated in integrin-binding peptide sequences to replicate extracellular cues [162], more recent experiments have demonstrated a viable lifespan of around 4 weeks in these tissues, with media formulation presumed to be a determining factor in hPCLS survival in culture [85].

There is enormous scope for enhanced reproducibility and replicability in the hPCLS model due to the large volume of slices generated from each donor [45, 63]. Prior to cryopreservation, cold storage of PCLS was explored as a possible method to maintain these tissues for later study [163, 164]. Initial efforts to cryopreserve PCLSs occurred in animals, with a 2014 study evaluating cryostorage of murine PCLS in 10% DMSO and Dulbecco’s modified Eagle medium (DMEM-F12) reporting a nearly 50% reduction in cellular metabolic activity post-thaw despite no significant loss in viability and preservation of airway contractility in response to chemical stimulation [132]. These findings were corroborated in a 2016 study also conducted in animal PCLSs, which further reinforced the ability of cryopreserved lung to appropriately respond to stimuli by analyzing PCLS response to zinc toxicity [157]. Another 2016 study using a similar preparation technique and the same cryopreservation medium in human PCLSs compared fresh and frozen slices from three donors, and found frozen tissue to have comparable phagocytic function in stimulated resident immune cells, comparable proliferative capacity in stimulated T cells, and comparable modulation of airway contractility in smooth muscle cells as a response to TAS2R agonists [82]. All of these studies utilized a DMSO-based (small-molecule) cryopreservative. With an appropriate protocol for cryopreservation, the high quantity of slices which can be generated from a single lobe of human lung thus gives this method immense potential for reproducibility in a single donor, and an equivalently high potential for replicability in a cryobank of multiple donors, overcoming the main shortcoming of ex vivo tissue models (Fig. 1) [163].

A 2023 study utilizing a proprietary cryopreservation medium provided the most detailed analysis to date on cryopreservation in hPCLSs. The authors determined there was no difference in viability, protein concentration, response to lipopolysaccharide (LPS), tissue structure, or surfactant production between fresh or frozen hPCLSs which were successfully cultured in DMEM-F12 supplemented with 1% insulin-transferrin-selenium for a period of 4 weeks [85]. The results of this study indicate that choice of cryopreservative and culture media are critical to effective utilization of cryobanks in the hPCLS model, suggesting the possibility that the thin nature of hPCLSs may allow for successful cryopreservation using DMSO, but side-effects associated with altered cell metabolism will be present unless a less toxic cryopreservative is used. Proper cryopreservation of hPCLSs thus greatly expands their utility as a tool for modeling human biology ex vivo by addressing the issue of tissue availability, and subsequently the time constraints limiting the reproducibility and replicability of this model. The usefulness of appropriate cryopreservation techniques to this end was confirmed in a 2024 study, where the ability of cryopreserved hPCLS to serve as a model of infection for viral pathogens such as severe acute respiratory syndrome coronavirus 2 (SARS-CoV-2) was explored as part of an analysis of the hPCLS platform in general [165]. Cryopreservation with non-toxic formulations thus holds great promise as a method for removing the greatest obstacles to the use of hPCLSs in studies of pulmonary biology.

### Explant tissue fragments

**Fig. 2:**
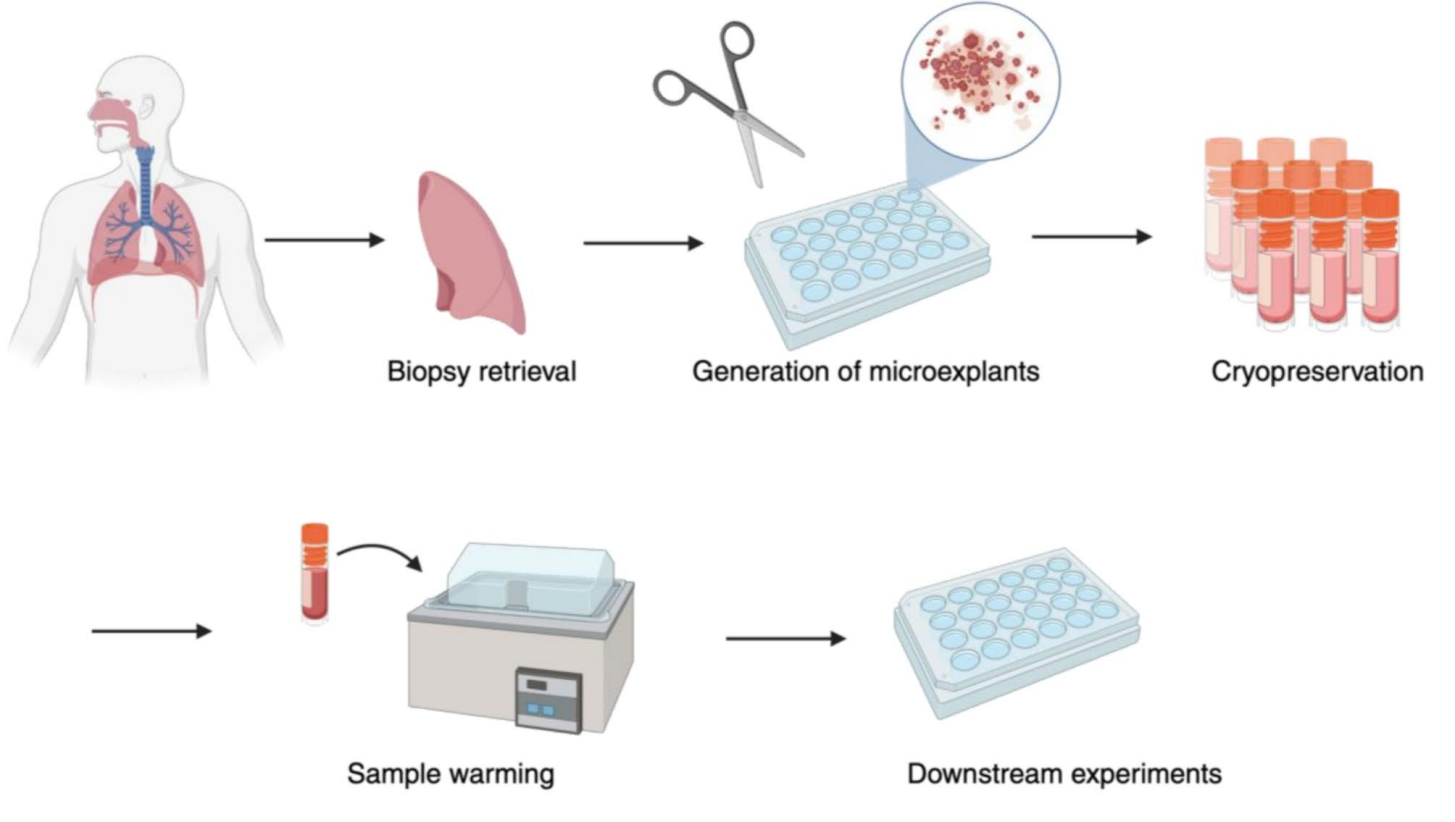
Diagram illustrating the sample preparation process for ex vivo lung microexplants. With the appropriate cryoprotectants, tissue microexplants can be up to approximately 0.5 cm^3^ in volume. Figure illustrations were generated in BioRender

Direct ex vivo culture of explanted lung tissue can be accomplished by culturing a whole explanted lung in its entirety or by culturing smaller fragments of dissected tissue [18, 47, 79]. While ex vivo lung perfusion systems that maintain a complete human lung for study are perhaps the method most faithful to human lung biology, this is also the most expensive, least reproducible, and least replicable way to culture human lung ex vivo, and removes available viable lungs from the transplant pool [166, 167]. Culturing dissected fragments of lung tissue from the same donor is therefore a preferable method to improve reproducibility, similar to the approach used in the generation of hPCLSs [168]. To create a 3D ex vivo tissue model of the lung parenchyma, the pleura and airways are removed from a fresh lobe of lung and the alveolar space is dissected into fragments of various dimensions which can then receive different experimental treatments [18, 47, 168, 169]. Ex vivo tissue fragments can vary in diameter from 1 mm or less (microexplants) [18] to nearly 10 cm [169], and carry the fully functional cellular diversity of the alveolar space in an accurate 3D structure [170] more relevant to whole lung tissue than that found in hPCLS or organoids [18]. Because the 3D architecture of the lung is the best preserved of all ex vivo models in this method, it is the most useful in studies where the spatial arrangement of lung cells is critical, such as those involving viral tropism [47, 170, 171], bacterial infections [172, 173], fibrogenesis [42], or cell migration [174]. Cryopreserved ex vivo tissue fragments can also function as the basis for the generation of decellularized tissue scaffolds [80] or organoids [175], both applications which will be discussed in later sections.

There are numerous experimental design considerations necessary when directly culturing ex vivo lung tissue fragments. Firstly, even in microexplants, the 3D nature of the tissue fragments makes the development of an air-liquid interface difficult due to the density of the tissue [18]. Reliable long-term (> 7 day) culture methods for ex vivo lung tissue fragments also remain elusive, though perfusion culture has been suggested as potentially extending the lifespan of these tissue fragments in other organs to a length comparable to that of hPCLSs [176]. While lung tissue fragments prepared in this manner retain resident innate and adaptive immune cells [18], the study of any phenomena involving the recruitment of immune cells from other sources to these tissues is challenging, though the incorporation of a hydrogel matrix as a scaffold to support these tissues does make the study of cell migration possible [172, 174, 177, 178]. Additionally, the higher concentration of developed ECM in these models compared to organoids or hPCLSs can obfuscate certain analyses contingent on the detection of molecules such as chemokines, some of which like CXCL8 might ultimately remain bound to the ECM throughout the culture period [18].

Similar to hPCLSs, cryopreservation of ex vivo tissue fragments in a cryobank can address the reproducibility and replicability shortcomings common to ex vivo lung culture models (Fig. 2) [31, 179]. Unlike in hPCLSs, however, the enhanced density of 3D ex vivo lung tissue fragments poses an additional challenge to their cryopreservation, as temperature changes throughout these fragments are not uniform [180] and small molecules are incapable of fully penetrating to the core of larger tissue fragments [18]. While smaller fragments (~ 5 mm^3^) may be adequately cryopreserved using small-molecule cryoprotectants such as DMSO [48, 77, 84], viability of larger fragments with this method is less favorable and the potential for undesired side-effects exists as noted in hPCLSs [79]. The successful cryopreservation of larger (up to ~ 0.5 cm^3^) ex vivo lung tissue fragments has been reported with the use of macromolecular cryoprotectants such as trehalose supplemented with surfactants, perfluorocarbons, and protease inhibitors [79], or the commercially available CryoSOFree [18, 174]. A 2014 study compared lung tissue fragments cryopreserved with a homebrew solution containing small molecule and macromolecular cryoprotectants and fresh lung tissue fragments below 0.5 cm^3^ from the same donors, and found similar protein profiles, cell viability, and tissue structure in healthy donors and those with idiopathic pulmonary fibrosis [79]. Although the exact mechanism for this improved viability remains unclear, based on the principles by which macromolecular cryoprotectants operate, we speculate it is possible that the extracellular control of osmotic pressure exerted by these molecules independent of their ability to reach the innermost cells of the tissue fragments is enough to prevent the formation of intracellular ice crystals deep within the core of the fragments and retain favorable viability.

Ex vivo culture of cryopreserved lung tissue fragments has been used to study viral infection of human lung tissue by SARS-CoV-2 and the migration of lung cancer cells along the alveolar surface [18, 174]. A 2019 study detailed the use of cryopreserved lung tissue fragments as microexplants to analyze the response to SARS-CoV-2 infection and characterized the cellular makeup of the microexplants, the transcription and translation of inflammatory mediators in response to drug treatment in the microexplants, and the viral titer of infected microexplants, determining that the microexplants maintained Type I and Type II alveolar epithelial cells, endothelial cells, T cells, and alveolar macrophages and monocytes throughout the cryopreservation process and demonstrated unimpaired cytokine production in response to infection. This information indicated that the microexplants were an appropriate model for the study of antiviral drugs, and the study further concluded that dexamethasone reduced SARS-CoV-2 viral titer in infected microexplants without affecting the production of inflammatory mediators [18]. A 2024 study analyzing cancer cell migration demonstrated that cells adherent to previously cryopreserved microexplants could also be quantified using intracellular dyes and flow cytometry [174].

### Decellularized lung

**Fig. 3:**
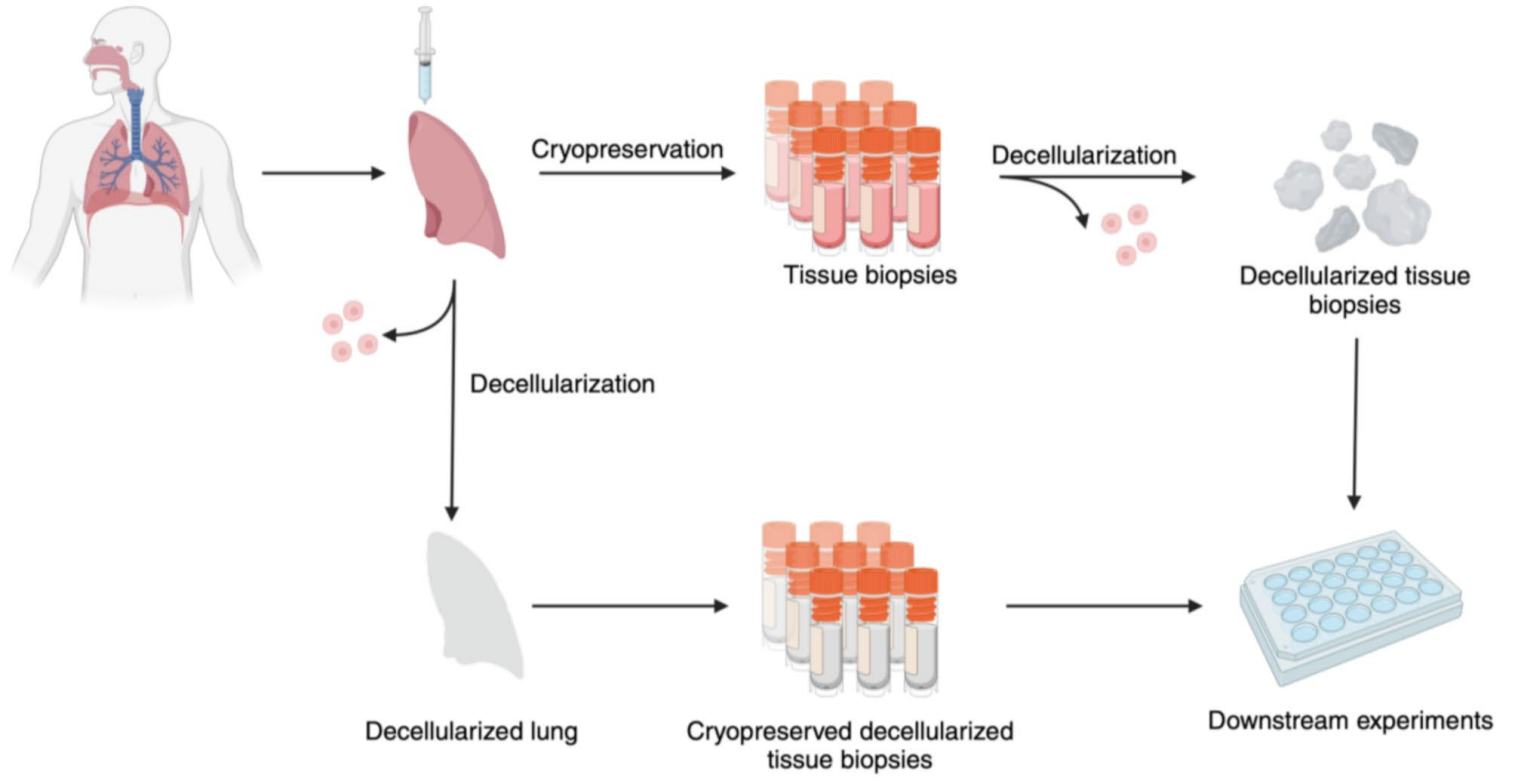
Diagram illustrating possible experimental protocols used for experiments involving decellularized lung. Tissues cryopreserved prior to decellularization can still be used in experiments as whole tissues or subjected to alternate decellularization protocols, while cryopreserved decellularized scaffolds are less adaptable for other protocols but a more rigorous model as all thawed scaffolds from a single donor would have undergone the same decellularization process. Figure illustrations were generated in BioRender.

Decellularization of lung lobes or whole lung from cadavers, followed by recellularization through seeding with stem cells from the target donor, was originally conceived as a method for generating viable lung tissue to be used in transplantation with minimal risk of rejection by the patient [181]. Currently, this application of this technique is limited by several factors, including the inability to completely remove all cells or cellular debris generated from the decellularization process without critically damaging the ECM [181, 182], damage potentially caused to collagens and proteoglycans in the ECM during the decellularization process [182, 183], the inability of seeded stem cells to properly form long-term functional vasculature [184], and the lack of innervation being key examples. Despite these shortcomings, the leftover ECM in the generation of decellularized lung scaffolds from animal or human tissue alike provides an excellent substrate onto which either cell lines or cryopreserved suspensions of primary cells can be seeded [185], a strategy not dissimilar to that used in the formation of organoids within the ECM-rich framework of Matrigel [186]. Decellularized human lungs are a particularly desirable basis for tissue engineering studies due to tissue-specific cues in the proteins, glycoproteins, and proteoglycans of the human ECM, collectively known as the matrisome [185, 187, 188]. Gentle decellularization of human lung tissue can thus be used as a source of 3D scaffolds [80, 185, 189] comprising matrisomes representative of disease states in afflictions such as asthma, chronic obstructive pulmonary disease, and idiopathic pulmonary fibrosis [78, 190, 191].

Decellularization of lungs is typically accomplished by perfusion of a detergent designed to separate cells from the lung ECM through either the airways, vasculature, or both [189, 192]. Depending on the decellularization method used, primary cells removed from lung tissue can be cryopreserved for later use [175] and seeded onto an acellular ECM scaffold [80]. For cryopreservation of these primary cells from any organ, small-molecule based cryopreservatives suitable for cell lines are appropriate and represent a well-covered ground in cryopreservation techniques [175, 193]. A central question in the cryopreservation of lung tissue expressly for decellularization is how the characteristics of the ECM are maintained [80]. Cryopreservation of small tissues as a source for small decellularized lung scaffolds has been successfully performed for laboratory experiments [185], and while cryopreservation of decellularized lung scaffolds themselves has not been well explored, cryopreserved decellularized pulmonary heart valves have been deployed clinically in allografts to avoid a deleterious immune response to lingering donor cells [194], suggesting promise for this approach. Decellularization could thus be performed prior to cryopreservation of tissues with the goal of cryopreserving scaffolds, or performed on tissues which have already been cryopreserved, as applicable to research objectives (Fig. 3). Construction of a cryobank with either approach would be useful in the utilization of tissue engineering techniques to study lung diseases in which critical factors may be related to the unique features of the matrisome in various donors [80, 185].

A 2023 study employed the snap-freezing and cryopreservation of lung tissue from healthy donors as a basis for a decellularized lung scaffold, onto which primary AECIIs from chronic obstructive pulmonary disease (COPD) donors were seeded in the study of ECM production and AECII behavior associated with the disease [80]. This study utilized a procedure in which peripheral lung tissue fragments of approximately 8 mm in diameter were flash-frozen and stored in a homebrew cryoprotectant containing 30% v/v glycerol, 30% v/v ethylene glycol, and 0.1 M sodium phosphate buffer, chosen for its ability to preserve the architecture of the lung tissue [185]. Post-thaw, the authors decellularized the tissue fragments before generating precision-cut lung slices of the remaining ECM and seeding them with primary AECIIs retrieved from a digestion of lung tissue of COPD patients. The authors used this model to successfully culture adherent AECIIs from healthy and COPD-afflicted donors for one week or more, and determined that there were few differences between seeded AECIIs from healthy donors and seeded AECIIs of COPD donors, implying the importance of the ECM composition in the behavior of these cells in COPD. The authors noted that the use of an acellular scaffold from COPD donors seeded with AECIIs from either healthy or COPD donors would provide a clearer picture as to the role of the ECM in this disease [80]. It is conceivable that macromolecular cryoprotectants appropriate for the preservation of lung tissue fragments would also allow cryostorage of lung tissue for the purpose of decellularization, therefore, further analysis of the effect of these cryopreservatives on the key signaling features of the ECM would prove useful in the construction of a cryobank for use in acellular scaffold generation, allowing the contributions of the ECM to lung disease to be more easily studied.

### Organoids

While originally referring to organ-like structures originating from tumors [195], the term “organoid” has since come to refer to organ-like structures which emerge from the 3D culture of stem cells within a gel matrix containing basement membrane components [69, 186, 196, 197]. The first animal lung organoids were cultured using murine fetal pulmonary cells in 2006 [198], and the first human lung organoids derived from AECIIs were produced in 2013 [199], followed by examples originating from pluripotent stem cells in 2015 [200]. Organoids are utilized in tissue engineering approaches to lung disease modeling [201, 202], and in the study of tissue development [203–205] and cancer [206, 207]. Lung organoids initiate from spheroid clusters of stem cells [200, 208], and with the correct signals from culture media or the surrounding matrix, can ultimately undergo a budding process through which structures resembling fetal tracheal [209], bronchial [210], and alveolar features can arise [205], with a thorough complement of intercellular interactions and gene expression critical to the emergence of these features available for analysis throughout [69, 70, 211]. Human lung organoids in particular can be derived from either adult lung stem cells [199], fetal lung stem cells [212], or induced pluripotent stem cells [200], allowing for the study of tissue-like structures in living patients with a minimal requirement for donor tissue [61]. Organoids are the ex vivo lung culture model for which there exists the most variety in cryopreservation strategies, as cryopreservation can be used to store the adult stem cells from which organoids originate [213], the tissues which are used as the source of these stem cells [18, 80], and the organoids themselves [214]. The cryopreservation of lung-sourced stem cells derived from donor tissue, the cryopreservation of lung organoids themselves, and the cryopreservation of small tissue expressly for the purpose of deriving organoids will be the focus of this section. A general discussion of the differentiation techniques and signaling cascades utilized to steer stem cells towards lung growth, as well as cryopreservation techniques particular to stem cells in general, are beyond the scope of this review.

By virtue of being stem cell-derived, organoids are highly customizable models of 3D ex vivo culture, with a great degree of heterogeneity in size and shape amongst cultures originating from the same cluster of cells [29]. Unlike the culture methods discussed above, gene editing with tools such as CRISPR/Cas9 is feasible in lung organoids [70] and has been used in studies of cancer [26, 61, 215], development [216], and idiopathic pulmonary fibrosis [217]. The matrix in which lung organoids reside can also be configured to accept an air-liquid interface [83]. Growth of human organoids requires a minimally invasive amount of tissue from donors, obtainable by biopsy [218], and can be used as a rapidly available, easily expandable, patient-matched testbed for therapeutic interventions in individualized disease conditions such as those found in cancer [61]. Lung cancer is a particularly useful utilization for organoid models of the lung, as the development process observed in cancer organoids can closely mirror patterns related to oncogenesis [219]. The broadly manipulable nature of organoids, however, can also be a limitation, as faithful reproduction of organ structures and disease characteristics requires a fine-tuned media and matrix formulation [70], which can potentially involve specific concentrations of numerous sensitive signal factors to develop organoids with the desired features [69]. Regardless of their origin, organoids are also essentially fetal in nature [205], meaning that they are anatomically simplified structures which do not possess the full complement of cells found in a developed organ, and the cells within them do not fully resemble those of a developed adult organ [220]. Organoids are thus currently incomplete models of adult tissue morphology, pending the discovery of other factors which regulate cellular differentiation [221]. Other models are therefore perhaps a better choice in the study of diseases which require the accurate simulation of relationships within complicated 3D structures such as alveoli.

**Fig. 4:**
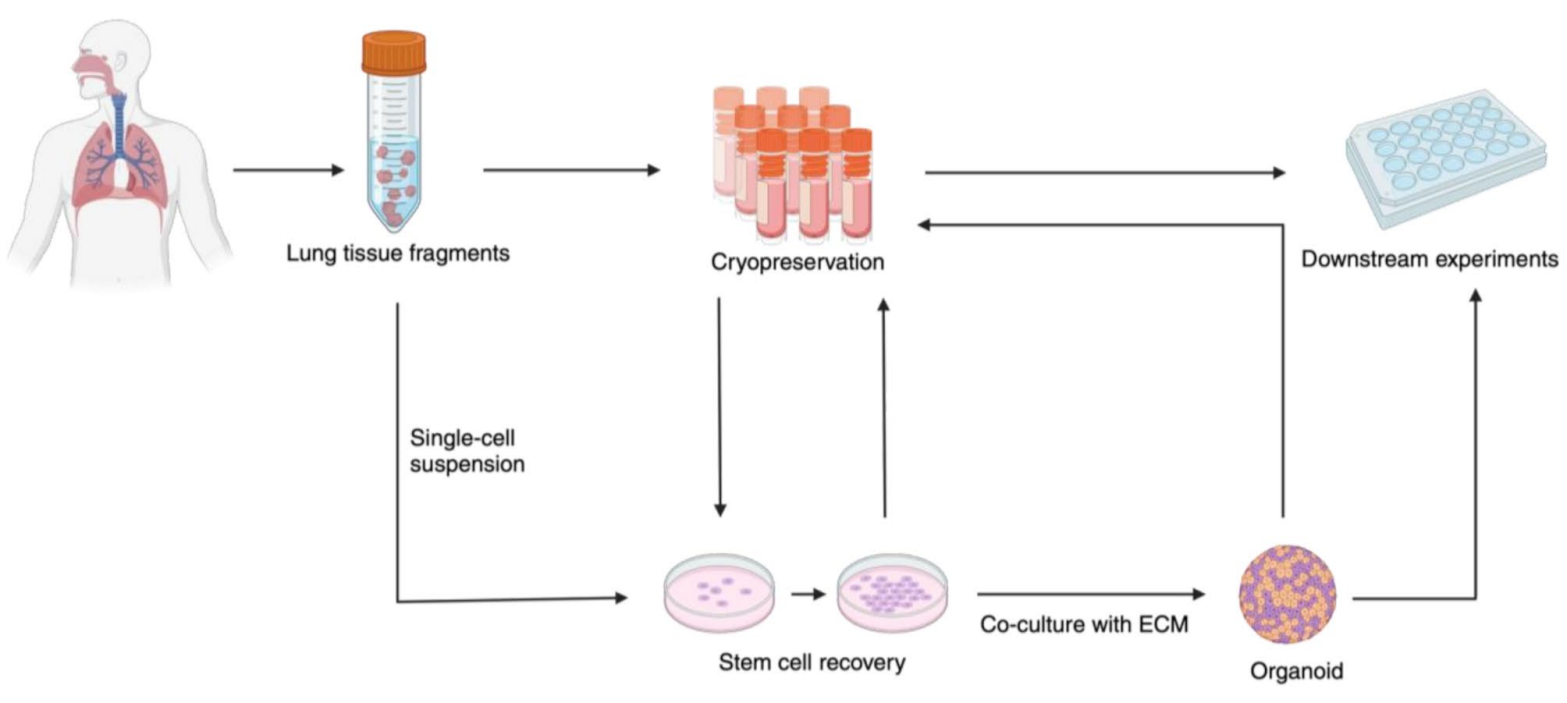
Diagram illustrating the cryopreservation of lung organoids in various stages of growth or preparation. Lung tissue fragments can be used to directly derive stem cells or instead cryopreserved and used later as a source of stem cells. These stem cells can either be cryopreserved or used to develop organoids, which can then either be used in experiments or cryopreserved themselves for convenient use at a later date. Figure illustrations were generated in BioRender

While it is possible to maintain live biobanks of serially passaged organoids [70], cryopreservation would provide the additional benefits of storage and transport to the use of such systems [214, 222] mitigate the effects of phenotypic drift [223], and can be incorporated into organoid preparation in numerous ways, all of which can be employed to form a cryobank [77, 212, 221]. Adult or fetal lung cells can be freed from fresh ex vivo tissue obtained from various donors and cryopreserved for later use in organoid generation [221]. In this instance, reproducibility is afforded by all thawed stem cells originating from the same donor and undergoing organoid growth in the presence of identical media factors and conditions for each experiment, whereas replicability is enabled through the sourcing of stem cells from multiple donors. Cryopreserved lung tissues themselves can also serve as the source of lung stem cells in this approach, which presents the advantage of allowing factors relating to organoid development to be tested as variables during organoid growth [48, 77, 83]. Alternatively, human lung organoids can be generated from fresh adult or fetal lung tissue and then themselves collected in a cryobank [212, 214, 222]. The benefit of this approach is that all banked organoids for each donor in this instance emerge from the same experimental conditions, providing superior reproducibility for studies not related to development, as this approach prevents the modification of factors associated with the growth stage of organoids (Fig. 4).

Several studies have characterized the effect of cryopreservation on tissue used as the basis for organoid models [48, 77, 212]. A 2017 paper utilizing lung organoids to study development employed a DMSO-based cryoprotectant and slow cooling to freeze human alveolar and bronchial organoid lines derived from either human fetal stem cells or adult lung tissue, in the identification of functional differences in the murine and human transcriptome of the lung distal tip epithelium. The authors cultured the organoids in Matrigel prior to dissolution of the matrix and eventual cryostorage, and reported no critical differences to the expression of *SOX* genes within the organoids or effects on the ability to incorporate plasmids into cryopreserved organoids post-thaw [212]. A 2021 study utilized DMEM supplemented with 10% DMSO and 20% fetal bovine serum (FBS) to slow-freeze parenchyma fragments approximately 4 mm in diameter prior to thawing and dissociation for organoid generation. The authors noted from single-cell RNA sequencing data that there were no significant changes in cellular identity, function, transcriptional, or epigenetic signatures as a result, but did not derive organoids from their thawed tissues [77]. Another 2021 study employing a similar method, but instead cryopreserving tissue fragments in CryoStor CS10, corroborated these findings before successfully forming organoids from cryopreserved tissue [48]. A 2023 protocol built upon these findings to establish a protocol by which cryopreserved tissues could be used as the basis for infection of lung organoids with SARS-CoV-2 in air-liquid interface culture, highlighting the promise of this method in the study of viral lung disease [83]. As they can be cryopreserved in multiple phases of their development with no apparent deleterious effects on critical genetic factors, organoids thus represent the most flexible ex vivo lung model in terms of cryopreservation protocols. Future analysis on the role of less-toxic cryoprotectants in the cryobanking of organoids, however, would confirm whether or not DMSO has significant effects on the developmental state represented in this model [105].

## Conclusions and future directions

Cryopreservation presents an answer for many of the challenges researchers face in the use of human lung tissue for ex vivo culture [48, 79, 179, 222]. Fresh human lung tissue is scarce, its procurement is difficult, and its viable window for experimental use in parallel with multiple donors is severely limited [66, 73]. Cryopreserved lung tissue is conveniently available, able to be used in various quantities as necessary, and able to be pooled with multiple donors [82–84]. These advantages are evident in the above studies, which have demonstrated the use of cryopreserved lung tissue in explant, lung slice, acellular, or organoid form to answer crucial questions about the biology of pulmonary diseases directly in humans. These new approaches to the study of human lung biology represent a movement closer to the goal of attaining greater fidelity to clinical settings in research studies, addressing the commonly cited problems of reproducibility and replicability in the use of fresh human lung tissue. Ex vivo models will continue to become more powerful as cryopreservation protocols evolve, methods to prolong the viability and translational relevance of cultured tissue improve, and strategies for synergistic utilization of each model become apparent.

Methods discovered to effectively cryopreserve and model lung tissue ex vivo could provide a template for the modeling of other organs ex vivo, providing differences in tissue density, structure, and cellular function are appropriately considered as variables affecting the cryopreservation process. Tissue characteristics, including porosity, cell density, and ECM structure, will affect the choice of cryoprotectant and method for adequately infusing frozen tissue with cryopreservative [87]. For smaller, less dense tissues in which diffusion readily occurs, formulations consisting primarily of small-molecule cryoprotectants will perform appropriately [82], while larger or dense sections of tissue will likely require a combination of small molecules and macromolecules to better control osmosis and ice crystal formation [18, 79]. In the case of large tissues specifically, where limited diffusion is an obstacle to fully protecting frozen tissue, new methods, techniques, or equipment designed to quickly perfuse fresh tissue with cryoprotectants and just as quickly flush it upon thawing may also improve viability. For any tissue, but particularly those in which local signaling cues are critical, the development of novel, biologically inert molecules capable of exerting the stabilizing effects necessary for survival of the freezing process without inducing unknown off-target effects on cellular biology will also alleviate existing concerns related to toxicity or undesirable alterations to transcription and translation routines after cryopreservation and thawing, such as occurs in some cell types with DMSO [104, 105]. These improvements in biological fidelity would not only apply to laboratory experiments involving tissue stored in cryobanks, but also to tissue stored for clinical purposes, highlighting effective cryopreservation strategies as a critical area for clinically focused research.

Despite the immense promise for human lung cryobanks, there is much that is yet to be explored about the effects of cryopreservation on human lung tissue. While the effects of specific cryoprotectants on viability, metabolism, transcription, and translation in lung tissue of various sizes has been evaluated [18, 48, 77, 79, 82, 85, 132, 212], there is much left to be determined about the effects of specific cryoprotectant formulations on the general biology of ex vivo human lung tissue. Indeed, there is no consensus method for stockpiling of lung tissue in cryobanks, and if not DMSO, many laboratories currently rely on homemade cryoprotectants designed to be specifically compatible with their research objectives. The specific effects of various cryopreservative formulations on certain disease states of the lung, such as idiopathic pulmonary fibrosis, are therefore elusive and present an obstacle to fully realizing the translational potential of ex vivo models. Studies which directly compare fresh tissue to frozen tissue remain the most effective benchmark in the observation of effects stemming from these individualized circumstances, and as long as validation against fresh tissue is necessary to confirm that cryopreserved tissue is safe to use in the testing of each unique hypothesis in the near term, the widespread benefit from the aforementioned advantages inherent in these ex vivo research methods will be underutilized due to lingering dependence on availability of rare fresh tissue. Large-scale -omics studies on tissues cryopreserved using the current most-studied cryopreservation methods might help to eliminate concerns about the effects of cryoprotectants on ex vivo lung biology [48], and further insight into the effect of cryopreservative formulation on the biology of ex vivo lung tissue is necessary to address this potential remaining hurdle in bridging the gap between the lab and the clinic.

Though cryopreservation is potentially a universally applicable solution to the reproducibility and replicability issues facing ex vivo lung models, it is still critical to consider the limitations inherent in each model when forming hypotheses for study. The aphorism of statistician George Box, “All models are wrong, some are useful,” [224] is especially relevant to the study of pulmonary disease in ex vivo models. However, as confined as these models may be in some areas, the direct use of human tissue holds the promise that these models will prove more useful than existing murine models which precede them. Human ex vivo models will never fully replace cell culture or animal models, but by complementing them, the translational gap can be narrowed and higher success rates for therapeutic development can eventually be achieved. Improvements in cryopreservation techniques and greater understanding of how they affect stored tissues will amplify the rate at which researchers are able to utilize ex vivo models in the study of disease, and subsequently accelerate our understanding of their shortcomings and fuel the discovery of methods which allow us to accommodate them. As the formation of cryobanks becomes commonplace, institutions for which the acquisition of human tissue is all but impossible will also be able to join the study of human ex vivo models, further enlarging the pool of data from which we can refine our knowledge of these models. Cryopreservation is therefore a fundamental tool in the utilization of ex vivo models and will ultimately play a key role in their broad adoption throughout the world of pulmonary biology.

## Data Availability

No datasets were generated or analysed during the current study.

## Abbreviations

3D: Three Dimensional
AECI: Type I Alveolar Epithelial Cell
AECII: Type II Alveolar Epithelial Cell
Cas: CRISPR Associated Protein
COPD: Chronic Obstructive Pulmonary Disease
CRISPR: Clustered Regularly Interspaced Short Palindromic Repeats
CXCL: C-X-C Motif Chemokine Ligand
DMEM: Dulbecco’s Modified Eagle Medium
DMSO: Dimethyl Sulfoxide
ECM: Extracellular Matrix
FBS: Fetal Bovine Serum
hPCLS: Human Precision-Cut Lung Slice
LPS: Lipopolysaccharide
PCLS: Precision-Cut Lung Slice
RNA: Ribonucleic Acid
SARS: CoV-Severe Acute Respiratory Symptom Coronavirus
SOX: SRY-Related HMG-Box Genes
TAS2R: Bitter Taste Receptor

## References

[1] Sanderson MJ. Exploring lung physiology in health and disease with lung slices. Pulm Pharmacol Ther [Internet]. 2011 Oct [cited 2024 Jun 24];24(5):452–65. Available from: https://www.ncbi.nlm.nih.gov/pmc/articles/PMC3168687/10.1016/j.pupt.2011.05.001 PMC3168687 21600999

[2] Matute-Bello G, Frevert CW, Martin TR. Animal models of acute lung injury. Am J Physiol - Lung Cell Mol Physiol [Internet]. 2008 Sep [cited 2024 Jun 24];295(3):L379–99. Available from: https://www.ncbi.nlm.nih.gov/pmc/articles/PMC2536793/10.1152/ajplung.00010.2008 PMC2536793 18621912

[3] Nichols JE, Niles JA, Vega SP, Argueta LB, Eastaway A, Cortiella J. Modeling the lung: Design and development of tissue engineered macro- and micro-physiologic lung models for research use. Exp Biol Med [Internet]. 2014 Sep 1 [cited 2024 Jun 24];239(9):1135–69. Available from: 10.1177/1535370214536679 10.1177/1535370214536679 24962174

[4] Miller AJ, Spence JR. In Vitro Models to Study Human Lung Development, Disease and Homeostasis. Physiology [Internet]. 2017 May [cited 2024 Jun 24];32(3):246–60. Available from: https://journals.physiology.org/doi/full/10.1152/physiol.00041.2016 10.1152/physiol.00041.2016 PMC6148341 28404740

[5] Kubo H. Molecular basis of lung tissue regeneration. Gen Thorac Cardiovasc Surg [Internet]. 2011 Apr 1 [cited 2024 Jun 24];59(4):231–44. Available from: 10.1007/s11748-010-0757-x 10.1007/s11748-010-0757-x 21484549

[6] Desai TJ, Brownfield DG, Krasnow MA. Alveolar progenitor and stem cells in lung development, renewal and cancer. Nature [Internet]. 2014 Mar 13 [cited 2024 Jun 24];507(7491):190–4. Available from: https://www.ncbi.nlm.nih.gov/pmc/articles/PMC4013278/10.1038/nature12930 PMC4013278 24499815

[7] Pardo-Saganta A, Law BM, Tata PR, Villoria J, Saez B, Mou H et al. Injury induces direct lineage segregation of functionally distinct airway basal stem/progenitor cell subpopulations. Cell Stem Cell [Internet]. 2015 Feb 5 [cited 2024 Jun 24];16(2):184–97. Available from: https://www.ncbi.nlm.nih.gov/pmc/articles/PMC4334442/10.1016/j.stem.2015.01.002 PMC4334442 25658372

[8] Sigmund CD, Carey RM, Appel L, Arnett D, Bosworth HB, Cushman WC et al. Report of the NHLBI Working Group on Hypertension: Barriers to Translation. Hypertens Dallas Tex 1979 [Internet]. 2020 Apr [cited 2024 Jun 24];75(4):902–17. Available from: https://www.ncbi.nlm.nih.gov/pmc/articles/PMC7067675/10.1161/HYPERTENSIONAHA.119.13887 PMC7067675 32063061

[9] O’Riordan TG, Smith V, Raghu G. Development of Novel Agents for Idiopathic Pulmonary Fibrosis: Progress in Target Selection and Clinical Trial Design. CHEST [Internet]. 2015 Oct 1 [cited 2024 Jun 24];148(4):1083–92. Available from: https://journal.chestnet.org/article/S0012-3692(15)50298-5/abstract 10.1378/chest.14-3218 26020856

[10] Mak IW, Evaniew N, Ghert M. Lost in translation: animal models and clinical trials in cancer treatment. Am J Transl Res [Internet]. 2014 Jan 15 [cited 2024 Jun 24];6(2):114–8. Available from: https://www.ncbi.nlm.nih.gov/pmc/articles/PMC3902221/PMC3902221 24489990

[11] Perrin S. Preclinical research: Make mouse studies work. Nature [Internet]. 2014 Mar [cited 2024 Jun 24];507(7493):423–5. Available from: https://www.nature.com/articles/507423a 10.1038/507423a 24678540

[12] Sun D, Gao W, Hu H, Zhou S. Why 90% of clinical drug development fails and how to improve it? Acta Pharm Sin B [Internet]. 2022 Jul 1 [cited 2024 Jun 24];12(7):3049–62. Available from: https://www.sciencedirect.com/science/article/pii/S2211383522000521 10.1016/j.apsb.2022.02.002 PMC9293739 35865092

[13] van de Wall G, van Timmermans HA, Ritskes-Hoitinga J, Bleich M, Leenaars A. C. Comparing translational success rates across medical research fields - A combined analysis of literature and clinical trial data. ALTEX - Altern Anim Exp [Internet]. 2023 Oct 17 [cited 2024 Jun 24];40(4):584–94. Available from: https://altex.org/index.php/altex/article/view/2539 10.14573/altex.2208261 37158378

[14] Frangogiannis NG. Why animal model studies are lost in translation. J Cardiovasc Aging [Internet]. 2022 Apr [cited 2024 Jun 24];2(2):22. Available from: https://www.ncbi.nlm.nih.gov/pmc/articles/PMC9052957/10.20517/jca.2022.10 PMC9052957 35497093

[15] Ott HC, Clippinger B, Conrad C, Schuetz C, Pomerantseva I, Ikonomou L et al. Regeneration and orthotopic transplantation of a bioartificial lung. Nat Med [Internet]. 2010 Aug [cited 2024 Jun 24];16(8):927–33. Available from: https://www.nature.com/articles/nm.2193 10.1038/nm.2193 20628374

[16] Kloxin AM, Lewis KJR, DeForest CA, Seedorf G, Tibbitt MW, Balasubramaniam V et al. Responsive culture platform to examine the influence of microenvironmental geometry on cell function in 3D. Integr Biol [Internet]. 2012 Nov 19 [cited 2024 Jun 24];4(12):1540–9. Available from: 10.1039/c2ib20212c 10.1039/c2ib20212c PMC3928973 23138879

[17] Gilpin SE, Wagner DE. Acellular human lung scaffolds to model lung disease and tissue regeneration. Eur Respir Rev [Internet]. 2018 Jun 6 [cited 2024 Jun 24];27(148):180021. Available from: https://www.ncbi.nlm.nih.gov/pmc/articles/PMC9488127/10.1183/16000617.0021-2018 PMC9488127 29875137

[18] Schaller MA, Sharma Y, Dupee Z, Nguyen D, Urueña J, Smolchek R et al. Ex vivo SARS-CoV-2 infection of human lung reveals heterogeneous host defense and therapeutic responses. JCI Insight [Internet]. [cited 2023 Aug 1];6(18):e148003. Available from: https://www.ncbi.nlm.nih.gov/pmc/articles/PMC8492301/10.1172/jci.insight.148003 PMC8492301 34357881

[19] Lang NJ, Gote-Schniering J, Porras-Gonzalez D, Yang L, De Sadeleer LJ, Jentzsch RC et al. Ex vivo tissue perturbations coupled to single-cell RNA-seq reveal multilineage cell circuit dynamics in human lung fibrogenesis. Sci Transl Med [Internet]. 2023 Dec 6 [cited 2024 Jun 24];15(725):eadh0908. Available from: https://www.science.org/doi/10.1126/scitranslmed.adh0908 10.1126/scitranslmed.adh0908 38055803

[20] Huh D, Matthews BD, Mammoto A, Montoya-Zavala M, Hsin HY, Ingber DE. Reconstituting organ-level lung functions on a chip. Science. 2010;328(5986):1662–8. 20576885 10.1126/science.1188302 PMC8335790

[21] Liu G, Särén L, Douglasson H, Zhou XH, Åberg PM, Ollerstam A et al. Precision cut lung slices: an ex vivo model for assessing the impact of immunomodulatory therapeutics on lung immune responses. Arch Toxicol [Internet]. 2021 Aug 1 [cited 2024 Jun 24];95(8):2871–7. Available from: 10.1007/s00204-021-03096-y 10.1007/s00204-021-03096-y 34191076

[22] Sikkema L, Ramírez-Suástegui C, Strobl DC, Gillett TE, Zappia L, Madissoon E et al. An integrated cell atlas of the lung in health and disease. Nat Med [Internet]. 2023 [cited 2024 Jul 9];29(6):1563–77. Available from: https://www.ncbi.nlm.nih.gov/pmc/articles/PMC10287567/10.1038/s41591-023-02327-2 PMC10287567 37291214

[23] Rosmark O, Ibáñez-Fonseca A, Thorsson J, Dellgren G, Hallgren O, Larsson Callerfelt AK et al. A tunable physiomimetic stretch system evaluated with precision cut lung slices and recellularized human lung scaffolds. Front Bioeng Biotechnol [Internet]. 2022 Oct 3 [cited 2025 Apr 23];10:995460. Available from: https://www.ncbi.nlm.nih.gov/pmc/articles/PMC9574011/10.3389/fbioe.2022.995460 PMC9574011 36263353

[24] Davidovich N, Huang J, Margulies SS. Reproducible uniform equibiaxial stretch of precision-cut lung slices. Am J Physiol - Lung Cell Mol Physiol [Internet]. 2013 Feb 15 [cited 2025 Apr 23];304(4):L210–20. Available from: https://www.ncbi.nlm.nih.gov/pmc/articles/PMC3567360/10.1152/ajplung.00224.2012 PMC3567360 23275624

[25] Xia JY, Zeng YF, Wu XJ, Xu F. Short-term ex vivo tissue culture models help study human lung infectionsA review. Medicine (Baltimore) [Internet]. 2023 Jan 6 [cited 2024 Jun 24];102(1):e32589. Available from: https://www.ncbi.nlm.nih.gov/pmc/articles/PMC9829290/10.1097/MD.0000000000032589 PMC9829290 36607848

[26] Taverna JA, Hung CN, Williams M, Williams R, Chen M, Kamali S et al. Ex vivo drug testing of patient-derived lung organoids to predict treatment responses for personalized medicine. Lung Cancer [Internet]. 2024 Apr 1 [cited 2024 Jun 24];190. Available from: https://www.lungcancerjournal.info/article/S0169-5002(24)00066-7/abstract 10.1016/j.lungcan.2024.107533 PMC12045304 38520909

[27] Wilkinson DC, Alva-Ornelas JA, Sucre JMS, Vijayaraj P, Durra A, Richardson W et al. Development of a Three-Dimensional Bioengineering Technology to Generate Lung Tissue for Personalized Disease Modeling. Stem Cells Transl Med [Internet]. 2017 Feb 1 [cited 2024 Jun 24];6(2):622–33. Available from: 10.5966/sctm.2016-0192 10.5966/sctm.2016-0192 PMC5442826 28191779

[28] Alsafadi HN, Staab-Weijnitz CA, Lehmann M, Lindner M, Peschel B, Königshoff M et al. An ex vivo model to induce early fibrosis-like changes in human precision-cut lung slices. Am J Physiol-Lung Cell Mol Physiol [Internet]. 2017 Jun [cited 2024 Jun 24];312(6):L896–902. Available from: 10.1152/ajplung.00084.2017 10.1152/ajplung.00084.2017 28314802

[29] Gkatzis K, Taghizadeh S, Huh D, Stainier DYR, Bellusci S. Use of three-dimensional organoids and lung-on-a-chip methods to study lung development, regeneration and disease. Eur Respir J [Internet]. 2018 Nov 1 [cited 2024 Jun 23];52(5). Available from: https://erj.ersjournals.com/content/52/5/1800876 10.1183/13993003.00876-2018 30262579

[30] Ubags NDJ, Alejandre Alcazar MA, Kallapur SG, Knapp S, Lanone S, Lloyd CM et al. Early origins of lung disease: towards an interdisciplinary approach. Eur Respir Rev [Internet]. 2020 Oct 1 [cited 2024 Jun 24];29(157):200191. Available from: https://www.ncbi.nlm.nih.gov/pmc/articles/PMC9488517/10.1183/16000617.0191-2020 PMC9488517 33004528

[31] Ross JT, Nesseler N, Lee JW, Ware LB, Matthay MA. The ex vivo human lung: research value for translational science. JCI Insight [Internet]. [cited 2024 Jun 24];4(11):e128833. Available from: https://www.ncbi.nlm.nih.gov/pmc/articles/PMC6629111/10.1172/jci.insight.128833 PMC6629111 31167972

[32] Hocke AC, Suttorp N, Hippenstiel S. Human lung ex vivo infection models. Cell Tissue Res [Internet]. 2017 [cited 2024 Jun 24];367(3):511–24. Available from: https://www.ncbi.nlm.nih.gov/pmc/articles/PMC7087833/10.1007/s00441-016-2546-z PMC7087833 27999962

[33] Zscheppang K, Berg J, Hedtrich S, Verheyen L, Wagner DE, Suttorp N et al. Human Pulmonary 3D Models For Translational Research. Biotechnol J [Internet]. 2018 Jan [cited 2024 Jun 24];13(1):1700341. Available from: https://www.ncbi.nlm.nih.gov/pmc/articles/PMC7161817/10.1002/biot.201700341 PMC7161817 28865134

[34] Burgstaller G, Gerckens M, Eickelberg O, Königshoff M. Decellularized Human Lung ScaffoldsScaffolds as Complex Three-Dimensional Tissue Culture Models to Study Functional Behavior of FibroblastsFibroblasts. In: Hinz B, Lagares D, editors. Myofibroblasts: Methods and Protocols [Internet]. New York, NY: Springer US; 2021 [cited 2024 Jun 24]. pp. 447–56. Available from: 10.1007/978-1-0716-1382-5_30

[35] Miller AJ, Dye BR, Ferrer-Torres D, Hill DR, Overeem AW, Shea LD et al. Generation of lung organoids from human pluripotent stem cells in vitro. Nat Protoc [Internet]. 2019 Feb [cited 2024 Jun 24];14(2):518–40. Available from: https://www.ncbi.nlm.nih.gov/pmc/articles/PMC6531049/10.1038/s41596-018-0104-8 PMC6531049 30664680

[36] Lang DS, Droemann D, Schultz H, Branscheid D, Martin C, Ressmeyer AR et al. A novel human ex vivo model for the analysis of molecular events during lung cancer chemotherapy. Respir Res [Internet]. 2007 [cited 2024 Jun 24];8(1):43. Available from: https://www.ncbi.nlm.nih.gov/pmc/articles/PMC1913052/10.1186/1465-9921-8-43 PMC1913052 17567922

[37] Yilmaz Y, Williams G, Walles M, Manevski N, Krähenbühl S, Camenisch G. Comparison of Rat and Human Pulmonary Metabolism Using Precision-cut Lung Slices (PCLS). Drug Metab Lett [Internet]. [cited 2024 Jun 25];13(1):53–63. Available from: https://www.eurekaselect.com/article/93865 10.2174/1872312812666181022114622 30345935

[38] Dekkers JF, Berkers G, Kruisselbrink E, Vonk A, de Jonge HR, Janssens HM et al. Characterizing responses to CFTR-modulating drugs using rectal organoids derived from subjects with cystic fibrosis. Sci Transl Med [Internet]. 2016 Jun 22 [cited 2024 Jun 25];8(344):344ra84-344ra84. Available from: https://www.science.org/doi/10.1126/scitranslmed.aad8278 10.1126/scitranslmed.aad8278 27334259

[39] Galasso M, Feld JJ, Watanabe Y, Pipkin M, Summers C, Ali A et al. Inactivating hepatitis C virus in donor lungs using light therapies during normothermic ex vivo lung perfusion. Nat Commun [Internet]. 2019 Jan 29 [cited 2024 Jun 24];10:481. Available from: https://www.ncbi.nlm.nih.gov/pmc/articles/PMC6351537/10.1038/s41467-018-08261-z PMC6351537 30696822

[40] Sachs N, Papaspyropoulos A, Zomer-van Ommen DD, Heo I, Böttinger L, Klay D et al. Long‐term expanding human airway organoids for disease modeling. EMBO J [Internet]. 2019 Feb 15 [cited 2024 Jun 24];38(4):e100300. Available from: https://www.ncbi.nlm.nih.gov/pmc/articles/PMC6376275/10.15252/embj.2018100300 PMC6376275 30643021

[41] Gretebeck LM, Subbarao K. Animal models for SARS and MERS coronaviruses. Curr Opin Virol [Internet]. 2015 Aug [cited 2024 Jun 24];13:123–9. Available from: https://www.ncbi.nlm.nih.gov/pmc/articles/PMC4550498/10.1016/j.coviro.2015.06.009 PMC4550498 26184451

[42] Roach KM, Sutcliffe A, Matthews L, Elliott G, Newby C, Amrani Y et al. A model of human lung fibrogenesis for the assessment of anti-fibrotic strategies in idiopathic pulmonary fibrosis. Sci Rep [Internet]. 2018 Jan 10 [cited 2024 Jun 24];8:342. Available from: https://www.ncbi.nlm.nih.gov/pmc/articles/PMC5762721/10.1038/s41598-017-18555-9 PMC5762721 29321510

[43] Banerjee SK, Huckuntod SD, Mills SD, Kurten RC, Pechous RD. Modeling Pneumonic Plague in Human Precision-Cut Lung Slices Highlights a Role for the Plasminogen Activator Protease in Facilitating Type 3 Secretion. Infect Immun [Internet]. 2019 Jul 23 [cited 2024 Jun 25];87(8):e00175-19. Available from: https://www.ncbi.nlm.nih.gov/pmc/articles/PMC6652753/10.1128/IAI.00175-19 PMC6652753 31085709

[44] Nizamoglu M, Joglekar MM, Almeida CR, Larsson Callerfelt AK, Dupin I, Guenat OT et al. Innovative three-dimensional models for understanding mechanisms underlying lung diseases: powerful tools for translational research. Eur Respir Rev [Internet]. 2023 Jul 26 [cited 2024 Jun 24];32(169):230042. Available from: https://www.ncbi.nlm.nih.gov/pmc/articles/PMC10369168/10.1183/16000617.0042-2023 PMC10369168 37495250

[45] Koziol-White C, Gebski E, Cao G, Panettieri RA. Precision cut lung slices: an integrated ex vivo model for studying lung physiology, pharmacology, disease pathogenesis and drug discovery. Respir Res [Internet]. 2024 [cited 2024 Jun 24];25:231. Available from: https://www.ncbi.nlm.nih.gov/pmc/articles/PMC11144351/10.1186/s12931-024-02855-6 PMC11144351 38824592

[46] Hui KPY, Ho JCW, Cheung MC, Ng KC, Ching RHH, Lai KL, et al. SARS-CoV-2 Omicron variant replication in human bronchus and lung ex vivo. Nature. 2022;603(7902):715–20. 35104836 10.1038/s41586-022-04479-6

[47] Weinheimer VK, Becher A, Tönnies M, Holland G, Knepper J, Bauer TT et al. Influenza A Viruses Target Type II Pneumocytes in the Human Lung. J Infect Dis [Internet]. 2012 Dec 1 [cited 2024 Jun 24];206(11):1685–94. Available from: https://www.ncbi.nlm.nih.gov/pmc/articles/PMC7107318/10.1093/infdis/jis455 PMC7107318 22829640

[48] Konda B, Mulay A, Yao C, Israely E, Beil S, Huynh CA et al. Cryobanking of Human Distal Lung Epithelial Cells for Preservation of Their Phenotypic and Functional Characteristics. Am J Respir Cell Mol Biol [Internet]. 2022 Dec [cited 2024 Jul 14];67(6):623–31. Available from: https://www.atsjournals.org/doi/10.1165/rcmb.2021-0507MA 10.1165/rcmb.2021-0507MA PMC12042124 36036918

[49] Cukierman E, Pankov R, Stevens DR, Yamada KM. Taking cell-matrix adhesions to the third dimension. Science. 2001;294(5547):1708–12. 11721053 10.1126/science.1064829

[50] Mondoñedo JR, Bartolák-Suki E, Bou Jawde S, Nelson K, Cao K, Sonnenberg A et al. A High-Throughput System for Cyclic Stretching of Precision-Cut Lung Slices During Acute Cigarette Smoke Extract Exposure. Front Physiol [Internet]. 2020 Jun 5 [cited 2025 Apr 23];11:566. Available from: https://www.ncbi.nlm.nih.gov/pmc/articles/PMC7326018/10.3389/fphys.2020.00566 PMC7326018 32655401

[51] Bagley DC, Russell T, Ortiz-Zapater E, Stinson S, Fox K, Redd PF et al. Bronchoconstriction damages airway epithelia by crowding-induced excess cell extrusion. Science [Internet]. 2024 Apr 5 [cited 2025 Apr 23];384(6691):66–73. Available from: https://www.science.org/doi/10.1126/science.adk2758 10.1126/science.adk2758 PMC7619646 38574138

[52] Shiraishi K, Shah PP, Morley MP, Loebel C, Santini GT, Katzen J et al. Biophysical forces mediated by respiration maintain lung alveolar epithelial cell fate. Cell [Internet]. 2023 Mar 30 [cited 2025 Apr 23];186(7):1478–1492.e15. Available from: https://www.ncbi.nlm.nih.gov/pmc/articles/PMC10065960/10.1016/j.cell.2023.02.010 PMC10065960 36870331

[53] Moriondo A, Marcozzi C, Bianchin F, Passi A, Boschetti F, Lattanzio S et al. Impact of respiratory pattern on lung mechanics and interstitial proteoglycans in spontaneously breathing anaesthetized healthy rats. Acta Physiol [Internet]. 2011 [cited 2025 Apr 23];203(2):331–41. Available from: https://onlinelibrary.wiley.com/doi/abs/10.1111/j.1748-1716.2011.02317.x 10.1111/j.1748-1716.2011.02317.x 21518268

[54] Liu CY, Chen YR, Mu HY, Huang JH. A Dynamic Breathing Lung Chip for Precise Evaluation of Inhaled Drug Efficacy and Airway Epithelial Responses. ACS Biomater Sci Eng [Internet]. 2024 Dec 1 [cited 2025 Apr 23];11(1):682–91. Available from: https://www.ncbi.nlm.nih.gov/pmc/articles/PMC11733924/10.1021/acsbiomaterials.4c01377 PMC11733924 39616618

[55] Mestas J, Hughes CCW. Of Mice and Not Men: Differences between Mouse and Human Immunology. J Immunol [Internet]. 2004 Mar 1 [cited 2024 Jun 24];172(5):2731–8. Available from: 10.4049/jimmunol.172.5.2731 10.4049/jimmunol.172.5.2731 14978070

[56] Parekh KR, Nawroth J, Pai A, Busch SM, Senger CN, Ryan AL. Stem cells and lung regeneration. Am J Physiol - Cell Physiol [Internet]. 2020 Oct 1 [cited 2024 Jun 25];319(4):C675–93. Available from: https://www.ncbi.nlm.nih.gov/pmc/articles/PMC7654650/10.1152/ajpcell.00036.2020 PMC7654650 32783658

[57] Miura T. Models of lung branching morphogenesis. J Biochem (Tokyo) [Internet]. 2015 Mar 1 [cited 2024 Jun 24];157(3):121–7. Available from: 10.1093/jb/mvu087 10.1093/jb/mvu087 25556243

[58] Lewin G, Hurtt ME. Pre- and Postnatal Lung Development: An Updated Species Comparison. Birth Defects Res [Internet]. 2017 [cited 2024 Jun 24];109(19):1519–39. Available from: https://onlinelibrary.wiley.com/doi/abs/10.1002/bdr2.1089 10.1002/bdr2.1089 28876535

[59] Cattaneo CM, Dijkstra KK, Fanchi LF, Kelderman S, Kaing S, van Rooij N et al. Tumor organoid– T cell co-culture systems. Nat Protoc [Internet]. 2020 Jan 1 [cited 2024 Jun 25];15(1):15–39. Available from: https://www.ncbi.nlm.nih.gov/pmc/articles/PMC7610702/10.1038/s41596-019-0232-9 PMC7610702 31853056

[60] Multidimensional Coculture System to Model Lung. Squamous Carcinoma Progression [Internet]. [cited 2024 Jul 14]. Available from: https://app.jove.com/t/60644/multidimensional-coculture-system-to-model-lung-squamous-carcinoma-progression 10.3791/60644 32250351

[61] Kim M, Mun H, Sung CO, Cho EJ, Jeon HJ, Chun SM et al. Patient-derived lung cancer organoids as in vitro cancer models for therapeutic screening. Nat Commun [Internet]. 2019 Sep 5 [cited 2024 Jul 10];10:3991. Available from: https://www.ncbi.nlm.nih.gov/pmc/articles/PMC6728380/10.1038/s41467-019-11867-6 PMC6728380 31488816

[62] Gao Q, Hartwig MG, Todd JL. Bridging the translation gap in cytomegalovirus therapeutics through ex vivo lung perfusion: Opportunities and challenges. J Heart Lung Transplant [Internet]. 2022 Mar 1 [cited 2024 Jul 15];41(3):298–9. Available from: https://www.jhltonline.org/article/S1053-2498(21)02615-2/abstract 10.1016/j.healun.2021.11.018 34969550

[63] Alsafadi HN, Uhl FE, Pineda RH, Bailey KE, Rojas M, Wagner DE et al. Applications and Approaches for Three-Dimensional Precision-Cut Lung Slices. Disease Modeling and Drug Discovery. Am J Respir Cell Mol Biol [Internet]. 2020 Jun [cited 2024 Jun 25];62(6):681. Available from: https://www.ncbi.nlm.nih.gov/pmc/articles/PMC7401444/10.1165/rcmb.2019-0276TR PMC7401444 31991090

[64] Soma S, Harriff MJ. Ex Vivo Lung Perfusion Provides New Insights into Human Lung-Resident Immune Cell Localization and Functional Interactions. Am J Respir Crit Care Med [Internet]. [cited 2024 Jun 25];203(10):1207–8. Available from: https://www.ncbi.nlm.nih.gov/pmc/articles/PMC8456468/10.1164/rccm.202012-4358ED PMC8456468 33357022

[65] Barkauskas CE, Chung MI, Fioret B, Gao X, Katsura H, Hogan BLM. Lung organoids: current uses and future promise. Dev Camb Engl [Internet]. 2017 Mar 15 [cited 2024 Jun 25];144(6):986–97. Available from: https://www.ncbi.nlm.nih.gov/pmc/articles/PMC5358104/10.1242/dev.140103 PMC5358104 28292845

[66] Liu G, Betts C, Cunoosamy DM, Åberg PM, Hornberg JJ, Sivars KB et al. Use of precision cut lung slices as a translational model for the study of lung biology. Respir Res [Internet]. 2019 [cited 2024 Jun 25];20:162. Available from: https://www.ncbi.nlm.nih.gov/pmc/articles/PMC6642541/10.1186/s12931-019-1131-x PMC6642541 31324219

[67] Hidiatov O, Gaupp A, Marini I, Pelzl L, Wagner M, Rigoni F et al. Characterization of Shear Stress Mediated Platelet Dysfunction: Data from an Ex Vivo Model for Extracorporeal Circulation and a Prospective Clinical Study. Thromb Haemost [Internet]. 2023 Apr [cited 2024 Sep 13];123(4):415–26. Available from: http://www.thieme-connect.de/DOI/DOI?10.1055/a-1988-3174. 10.1055/a-1988-3174 36442804

[68] Eltanahy AM, Franco C, Jeyaraj P, Goswami S, Hughes E, Gonzales AL. Ex-Vivo Ocular Perfusion Model to Study Vascular Physiology in the Mouse Eye. Exp Eye Res [Internet]. 2023 Aug [cited 2024 Sep 13];233:109543. Available from: https://www.ncbi.nlm.nih.gov/pmc/articles/PMC10637262/10.1016/j.exer.2023.109543 PMC10637262 37390954

[69] Vazquez-Armendariz AI, Tata PR. Recent advances in lung organoid development and applications in disease modeling. J Clin Invest [Internet]. [cited 2024 Jul 9];133(22):e170500. Available from: https://www.ncbi.nlm.nih.gov/pmc/articles/PMC10645385/10.1172/JCI170500 PMC10645385 37966116

[70] Ebisudani T, Hamamoto J, Togasaki K, Mitsuishi A, Sugihara K, Shinozaki T et al. Genotype-phenotype mapping of a patient-derived lung cancer organoid biobank identifies NKX2-1-defined Wnt dependency in lung adenocarcinoma. Cell Rep [Internet]. 2023 Mar 28 [cited 2024 Jul 9];42(3). Available from: https://www.cell.com/cell-reports/abstract/S2211-1247(23)00223-1 10.1016/j.celrep.2023.112212 36870059

[71] Leithner K, Wohlkoenig C, Stacher E, Lindenmann J, Hofmann NA, Gallé B et al. Hypoxia increases membrane metallo-endopeptidase expression in a novel lung cancer ex vivo model– role of tumor stroma cells. BMC Cancer [Internet]. 2014 Jan 25 [cited 2024 Sep 13];14:40. Available from: https://www.ncbi.nlm.nih.gov/pmc/articles/PMC3905926/10.1186/1471-2407-14-40 PMC3905926 24460801

[72] Tawhai MH, Bates JHT. Multi-scale lung modeling. J Appl Physiol [Internet]. 2011 May [cited 2024 Jun 24];110(5):1466–72. Available from: https://www.ncbi.nlm.nih.gov/pmc/articles/PMC3098667/10.1152/japplphysiol.01289.2010 PMC3098667 21292842

[73] Grizzle WE, Bell WC, Sexton KC. Issues in collecting, processing and storing human tissues and associated information to support biomedical research. Cancer Biomark Sect Dis Markers [Internet]. 2010 [cited 2024 Jun 23];9(1–6):531–49. Available from: https://www.ncbi.nlm.nih.gov/pmc/articles/PMC3445033/10.3233/CBM-2011-0183 PMC3445033 22112494

[74] Lehmann M, Krishnan R, Sucre J, Kulkarni HS, Pineda RH, Anderson C et al. Precision-Cut Lung Slices: Emerging Tools for Preclinical and Translational Lung Research: An Official American Thoracic Society Workshop Report. Am J Respir Cell Mol Biol [Internet]. [cited 2025 Apr 24];72(1):16–31. Available from: https://www.ncbi.nlm.nih.gov/pmc/articles/PMC11707673/10.1165/rcmb.2024-0479ST PMC11707673 39499861

[75] Travaglini KJ, Nabhan AN, Penland L, Sinha R, Gillich A, Sit RV et al. A molecular cell atlas of the human lung from single-cell RNA sequencing. Nature [Internet]. 2020 Nov [cited 2024 Jul 30];587(7835):619–25. Available from: https://www.nature.com/articles/s41586-020-2922-4 10.1038/s41586-020-2922-4 PMC7704697 33208946

[76] Winkler J, Herzog EL. Biobanking for Pulmonary, Critical Care, and Sleep Medicine. In: Gomez JL, Himes BE, Kaminski N, editors. Precision in Pulmonary, Critical Care, and Sleep Medicine: A Clinical and Research Guide [Internet]. Cham: Springer International Publishing; 2020 [cited 2024 Jun 23]. pp. 117–30. Available from: 10.1007/978-3-030-31507-8_9

[77] Llamazares-Prada M, Espinet E, Mijošek V, Schwartz U, Lutsik P, Tamas R et al. Versatile workflow for cell type–resolved transcriptional and epigenetic profiles from cryopreserved human lung. JCI Insight [Internet]. [cited 2024 Jun 25];6(6):e140443. Available from: https://www.ncbi.nlm.nih.gov/pmc/articles/PMC8026197/10.1172/jci.insight.140443 PMC8026197 33630765

[78] Sava P, Ramanathan A, Dobronyi A, Peng X, Sun H, Ledesma-Mendoza A et al. Human pericytes adopt myofibroblast properties in the microenvironment of the IPF lung. JCI Insight [Internet]. [cited 2024 Jul 8];2(24):e96352. Available from: https://www.ncbi.nlm.nih.gov/pmc/articles/PMC5752282/10.1172/jci.insight.96352 PMC5752282 29263297

[79] Baatz JE, Newton DA, Riemer EC, Denlinger CE, Jones EE, Drake RR et al. Cryopreservation of Viable Human Lung Tissue for Versatile Post-thaw Analyses and Culture. Vivo Athens Greece [Internet]. 2014 [cited 2024 Jul 1];28(4):411–23. Available from: https://www.ncbi.nlm.nih.gov/pmc/articles/PMC5937261/PMC5937261 24982205

[80] Rosmark O, Kadefors M, Dellgren G, Karlsson C, Ericsson A, Lindstedt S et al. Alveolar epithelial cells are competent producers of interstitial extracellular matrix with disease relevant plasticity in a human in vitro 3D model. Sci Rep [Internet]. 2023 May 31 [cited 2024 Jul 7];13:8801. Available from: https://www.ncbi.nlm.nih.gov/pmc/articles/PMC10232446/10.1038/s41598-023-35011-z PMC10232446 37258541

[81] National Academies of Sciences E, Affairs P, Committee on Science G, Information E, on RD B, Sciences DonE, Statistics P et al. C on A and T,. Understanding Reproducibility and Replicability. In: Reproducibility and Replicability in Science [Internet]. National Academies Press (US); 2019 [cited 2024 Jul 14]. Available from: https://www.ncbi.nlm.nih.gov/books/NBK547546/

[82] Bai Y, Krishnamoorthy N, Patel KR, Rosas I, Sanderson MJ, Ai X. Cryopreserved Human Precision-Cut Lung Slices as a Bioassay for Live Tissue Banking. A Viability Study of Bronchodilation with Bitter-Taste Receptor Agonists. Am J Respir Cell Mol Biol [Internet]. 2016 May [cited 2024 Jun 19];54(5):656–63. Available from: https://www.ncbi.nlm.nih.gov/pmc/articles/PMC4942196/10.1165/rcmb.2015-0290MA PMC4942196 26550921

[83] Castaneda DC, Jangra S, Yurieva M, Martinek J, Callender M, Coxe M et al. Protocol for establishing primary human lung organoid-derived air-liquid interface cultures from cryopreserved human lung tissue. STAR Protoc [Internet]. 2023 Nov 21 [cited 2024 Jun 25];4(4):102735. Available from: https://www.ncbi.nlm.nih.gov/pmc/articles/PMC10696416/10.1016/j.xpro.2023.102735 PMC10696416 37991921

[84] Roth M, Solèr M, Hornung M, Emmons LR, Stulz P, Perruchoud AP. Cell cultures from cryopreserved human lung tissue. Tissue Cell [Internet]. 1992 Jan 1 [cited 2024 Jun 25];24(4):455–9. Available from: https://www.sciencedirect.com/science/article/pii/004081669290061B 10.1016/0040-8166(92)90061-b 1440571

[85] Patel VS, Amin K, Wahab A, Marimoutou M, Ukishima L, Alvarez J, et al. Cryopreserved human precision-cut lung slices provide an immune competent pulmonary test system for on-demand use and long-term cultures. Toxicol Sci Off J Soc Toxicol. 2023;191(2):253–65. 10.1093/toxsci/kfac136 PMC9936202 36617185

[86] Pegg DE. Principles of Cryopreservation. In: Wolkers WF, Oldenhof H, editors. Cryopreservation and Freeze-Drying Protocols [Internet]. New York, NY: Springer; 2015 [cited 2024 Jul 1]. pp. 3–19. Available from: 10.1007/978-1-4939-2193-5_1

[87] Murray KA, Gibson MI. Chemical approaches to cryopreservation. Nat Rev Chem [Internet]. 2022 Aug [cited 2024 Jun 27];6(8):579–93. Available from: https://www.nature.com/articles/s41570-022-00407-4 10.1038/s41570-022-00407-4 PMC9294745 35875681

[88] Mazur P, Leibo SP, Chu EHY. A two-factor hypothesis of freezing injury: Evidence from Chinese hamster tissue-culture cells. Exp Cell Res [Internet]. 1972 Apr 1 [cited 2024 Jun 26];71(2):345–55. Available from: https://www.sciencedirect.com/science/article/pii/0014482772903035 10.1016/0014-4827(72)90303-5 5045639

[89] Mazur P, Schmidt JJ. Interactions of cooling velocity, temperature, and warming velocity on the survival of frozen and thawed yeast. Cryobiology [Internet]. 1968 Jul 1 [cited 2024 Jun 26];5(1):1–17. Available from: https://www.sciencedirect.com/science/article/pii/S0011224068801385 10.1016/s0011-2240(68)80138-5 5760041

[90] Ishiguro H, Rubinsky B. Mechanical Interactions between Ice Crystals and Red Blood Cells during Directional Solidification. Cryobiology [Internet]. 1994 Oct 1 [cited 2024 Jun 26];31(5):483–500. Available from: https://www.sciencedirect.com/science/article/pii/S0011224084710595 10.1006/cryo.1994.1059 7988158

[91] Toner M, Cravalho EG, Karel M, Armant DR. Cryomicroscopic analysis of intracellular ice formation during freezing of mouse oocytes without cryoadditives. Cryobiology [Internet]. 1991 Feb 1 [cited 2024 Jun 26];28(1):55–71. Available from: https://www.sciencedirect.com/science/article/pii/001122409190008C 10.1016/0011-2240(91)90008-c 2015761

[92] Gao Y, Qi H, Zhang L. Advances in Antifreeze Molecules: From Design and Mechanisms to Applications. Ind Eng Chem Res [Internet]. 2023 May 24 [cited 2024 Jun 27];62(20):7839–58. Available from: 10.1021/acs.iecr.3c00690

[93] Lomba L, García CB, Benito L, Sangüesa E, Santander S, Zuriaga E. Advances in Cryopreservatives: Exploring Safer Alternatives. ACS Biomater Sci Eng [Internet]. 2024 Jan 8 [cited 2024 Jun 27];10(1):178–90. Available from: 10.1021/acsbiomaterials.3c00859 10.1021/acsbiomaterials.3c00859 38141007

[94] Mori S, Choi J, Devireddy RV, Bischof JC. Calorimetric measurement of water transport and intracellular ice formation during freezing in cell suspensions. Cryobiology [Internet]. 2012 Dec 1 [cited 2024 Jun 26];65(3):242–55. Available from: https://www.sciencedirect.com/science/article/pii/S0011224012002027 10.1016/j.cryobiol.2012.06.010 22863747

[95] Pi CH, Yu G, Petersen A, Hubel A. Characterizing the sweet spot for the preservation of a T-cell line using osmolytes. Sci Rep [Internet]. 2018 Nov 1 [cited 2024 Jun 26];8:16223. Available from: https://www.ncbi.nlm.nih.gov/pmc/articles/PMC6212455/10.1038/s41598-018-34638-7 PMC6212455 30385865

[96] Han B, Bischof JC. Direct cell injury associated with eutectic crystallization during freezing. Cryobiology [Internet]. 2004 Feb 1 [cited 2024 Jun 26];48(1):8–21. Available from: https://www.sciencedirect.com/science/article/pii/S0011224003001123 10.1016/j.cryobiol.2003.11.002 14969678

[97] Stubbs C, Bailey TL, Murray K, Gibson MI. Polyampholytes as Emerging Macromolecular Cryoprotectants. Biomacromolecules [Internet]. 2020 Jan 13 [cited 2023 Feb 22];21(1):7–17. Available from: 10.1021/acs.biomac.9b01053 10.1021/acs.biomac.9b01053 PMC6960013 31418266

[98] Klbik I, Čechová K, Milovská S, Rusnák J, Vlasáč J, Melicherčík M et al. Cryoprotective Mechanism of DMSO Induced by the Inhibitory Effect on Eutectic NaCl Crystallization. J Phys Chem Lett [Internet]. 2022 Dec 8 [cited 2024 Jun 27];13(48):11153–9. Available from: 10.1021/acs.jpclett.2c03003 10.1021/acs.jpclett.2c03003 36442496

[99] Chian RC, Wang Y, Li YR. Oocyte vitrification: advances, progress and future goals. J Assist Reprod Genet [Internet]. 2014 Apr [cited 2024 Jun 28];31(4):411–20. Available from: https://www.ncbi.nlm.nih.gov/pmc/articles/PMC3969469/10.1007/s10815-014-0180-9 PMC3969469 24477781

[100] Santis LD, Coticchio G. Theoretical and experimental basis of slow freezing. Reprod Biomed Online [Internet]. 2011 Feb 1 [cited 2024 Jun 28];22(2):125–32. Available from: https://www.rbmojournal.com/article/S1472-6483(10)00707-8/fulltext 10.1016/j.rbmo.2010.10.012 21237713

[101] Rajan R, Hayashi F, Nagashima T, Matsumura K. Toward a Molecular Understanding of the Mechanism of Cryopreservation by Polyampholytes: Cell Membrane Interactions and Hydrophobicity. Biomacromolecules [Internet]. 2016 May 9 [cited 2023 Feb 22];17(5):1882–93. Available from: 10.1021/acs.biomac.6b00343 10.1021/acs.biomac.6b00343 27077533

[102] Polge C, Smith AU, Parkes AS. Revival of Spermatozoa after Vitrification and Dehydration at Low Temperatures. Nature [Internet]. 1949 Oct [cited 2024 Jun 26];164(4172):666–666. Available from: https://www.nature.com/articles/164666a0 10.1038/164666a0 18143360

[103] Lovelock JE, Bishop MWH. Prevention of Freezing Damage to Living Cells by Dimethyl Sulphoxide. Nature [Internet]. 1959 May [cited 2024 Jun 26];183(4672):1394–5. Available from: https://www.nature.com/articles/1831394a0 10.1038/1831394a0 13657132

[104] Timm M, Saaby L, Moesby L, Hansen EW. Considerations regarding use of solvents in in vitro cell based assays. Cytotechnology [Internet]. 2013 Oct 1 [cited 2024 Jun 27];65(5):887–94. Available from: 10.1007/s10616-012-9530-6 10.1007/s10616-012-9530-6 PMC3967611 23328992

[105] Pal R, Mamidi MK, Das AK, Bhonde R. Diverse effects of dimethyl sulfoxide (DMSO) on the differentiation potential of human embryonic stem cells. Arch Toxicol [Internet]. 2012 Apr 1 [cited 2024 Jun 27];86(4):651–61. Available from: 10.1007/s00204-011-0782-2 10.1007/s00204-011-0782-2 22105179

[106] Awan M, Buriak I, Fleck R, Fuller B, Goltsev A, Kerby J et al. Dimethyl Sulfoxide: A Central Player Since the Dawn of Cryobiology, is Efficacy Balanced by Toxicity? Regen Med [Internet]. 2020 Mar 1 [cited 2024 Jun 27];15(3):1463–91. Available from: 10.2217/rme-2019-0145 10.2217/rme-2019-0145 32342730

[107] Kang MH, You SY, Hong K, Kim JH. DMSO impairs the transcriptional program for maternal-to-embryonic transition by altering histone acetylation. Biomaterials [Internet]. 2020 Feb 1 [cited 2024 Jun 27];230:119604. Available from: https://www.sciencedirect.com/science/article/pii/S0142961219307033 10.1016/j.biomaterials.2019.119604 31761489

[108] Verheijen M, Lienhard M, Schrooders Y, Clayton O, Nudischer R, Boerno S et al. DMSO induces drastic changes in human cellular processes and epigenetic landscape in vitro. Sci Rep [Internet]. 2019 Mar 15 [cited 2024 Jun 27];9:4641. Available from: https://www.ncbi.nlm.nih.gov/pmc/articles/PMC6420634/10.1038/s41598-019-40660-0 PMC6420634 30874586

[109] Yao X, Matosevic S. Cryopreservation of NK and T Cells Without DMSO for Adoptive Cell-Based Immunotherapy. BioDrugs [Internet]. 2021 Sep 1 [cited 2024 Jun 27];35(5):529–45. Available from: 10.1007/s40259-021-00494-7 10.1007/s40259-021-00494-7 PMC12376086 34427899

[110] Mohammed L, Marquez-Curtis LA, Elliott JAW. Cryopreservation of human cerebral microvascular endothelial cells with glycerol. Cryobiology [Internet]. 2023 Dec 1 [cited 2024 Jun 27];113:104551. Available from: https://www.sciencedirect.com/science/article/pii/S0011224023000500 10.1016/j.cryobiol.2023.104551 37328025

[111] Wolkers WF, Oldenhof H, editors. Cryopreservation and Freeze-Drying Protocols [Internet]. New York NY: Springer. 2015 [cited 2024 Jun 27]. (Methods in Molecular Biology; vol. 1257). Available from: https://link.springer.com/10.1007/978-1-4939-2193-5

[112] Bhatnagar BS, Bogner RH, Pikal MJ. Protein Stability During Freezing: Separation of Stresses and Mechanisms of Protein Stabilization. Pharm Dev Technol [Internet]. 2007 Jan 1 [cited 2024 Jun 27];12(5):505–23. Available from: 10.1080/10837450701481157 10.1080/10837450701481157 17963151

[113] Braudeau C, Salabert-Le Guen N, Chevreuil J, Rimbert M, Martin JC, Josien R. An easy and reliable whole blood freezing method for flow cytometry immuno-phenotyping and functional analyses. Cytometry B Clin Cytom [Internet]. 2021 [cited 2025 Apr 24];100(6):652–65. Available from: https://onlinelibrary.wiley.com/doi/abs/10.1002/cyto.b.21994 10.1002/cyto.b.21994 33544978

[114] Naaldijk Y, Johnson AA, Friedrich-Stöckigt A, Stolzing A. Cryopreservation of dermal fibroblasts and keratinocytes in hydroxyethyl starch–based cryoprotectants. BMC Biotechnol [Internet]. 2016 Dec 1 [cited 2025 Apr 24];16:85. Available from: https://www.ncbi.nlm.nih.gov/pmc/articles/PMC5131400/10.1186/s12896-016-0315-4 PMC5131400 27903244

[115] Murray KA, Gibson MI. Post-Thaw Culture and Measurement of Total Cell Recovery Is Crucial in the Evaluation of New Macromolecular Cryoprotectants. Biomacromolecules [Internet]. 2020 Jul 13 [cited 2024 Jun 27];21(7):2864–73. Available from: 10.1021/acs.biomac.0c00591 10.1021/acs.biomac.0c00591 PMC7362331 32501710

[116] Konov KB, Isaev NP, Dzuba SA. Low-Temperature Molecular Motions in Lipid Bilayers in the Presence of Sugars: Insights into Cryoprotective Mechanisms. J Phys Chem B [Internet]. 2014 Oct 30 [cited 2024 Jun 28];118(43):12478–85. Available from: 10.1021/jp508312n 10.1021/jp508312n 25296133

[117] Gertrudes A, Craveiro R, Eltayari Z, Reis RL, Paiva A, Duarte ARC. How Do Animals Survive Extreme Temperature Amplitudes? The Role of Natural Deep Eutectic Solvents. ACS Sustain Chem Eng [Internet]. 2017 Nov 6 [cited 2024 Jun 28];5(11):9542–53. Available from: 10.1021/acssuschemeng.7b01707

[118] Sømme L, Meier T. Cold tolerance in Tardigrada from Dronning Maud Land, Antarctica. Polar Biol [Internet]. 1995 Feb 1 [cited 2024 Sep 13];15(3):221–4. Available from: 10.1007/BF00239062

[119] Koštál V, Doležal P, Rozsypal J, Moravcová M, Zahradníčková H, Šimek P. Physiological and biochemical analysis of overwintering and cold tolerance in two Central European populations of the spruce bark beetle, *Ips typographus*. J Insect Physiol [Internet]. 2011 Aug 1 [cited 2024 Sep 13];57(8):1136–46. Available from: https://www.sciencedirect.com/science/article/pii/S0022191011000795 10.1016/j.jinsphys.2011.03.011 21420974

[120] Storey KB, Storey JM. Biochemical adaption for freezing tolerance in the wood frog,Rana sylvatica. J Comp Physiol B [Internet]. 1984 Jan 1 [cited 2024 Sep 13];155(1):29–36. Available from: 10.1007/BF00688788

[121] Hirata T, Yokomise H, Fukuse T, Muro K, Inui K, Yagi K et al. Effects of Trehalose in Preservation of Canine Lung for Transplants. Thorac Cardiovasc Surg [Internet]. 1993 Feb [cited 2024 Jun 28];41(1):59–63. Available from: http://www.thieme-connect.de/DOI/DOI?10.1055/s-2007-1013822. 10.1055/s-2007-1013822 8367858

[122] Katenz E, Vondran FWR, Schwartlander R, Pless G, Gong X, Cheng X et al. Cryopreservation of primary human hepatocytes: The benefit of trehalose as an additional cryoprotective agent. Liver Transpl [Internet]. 2007 [cited 2024 Jun 28];13(1):38–45. Available from: https://onlinelibrary.wiley.com/doi/abs/10.1002/lt.20921 10.1002/lt.20921 17154395

[123] Hu Y, Liu X, Zhang W, Chen J, Chen X, Tan S. Inulin Can Improve Red Blood Cell Cryopreservation by Promoting Vitrification, Stabilizing Cell Membranes, and Inhibiting Ice Recrystallization. ACS Biomater Sci Eng [Internet]. 2024 Feb 12 [cited 2024 Jun 28];10(2):851–62. Available from: 10.1021/acsbiomaterials.3c01463 10.1021/acsbiomaterials.3c01463 38176101

[124] Matsumura K, Hayashi F, Nagashima T, Rajan R, Hyon SH. Molecular mechanisms of cell cryopreservation with polyampholytes studied by solid-state NMR. Commun Mater [Internet]. 2021 Feb 9 [cited 2024 Jun 28];2(1):1–12. Available from: https://www.nature.com/articles/s43246-021-00118-1

[125] Graether SP, Kuiper MJ, Gagné SM, Walker VK, Jia Z, Sykes BD et al. β-Helix structure and ice-binding properties of a hyperactive antifreeze protein from an insect. Nature [Internet]. 2000 Jul [cited 2024 Jun 28];406(6793):325–8. Available from: https://www.nature.com/articles/35018610 10.1038/35018610 10917537

[126] Hauet T, Eugene M. A new approach in organ preservation: potential role of new polymers. Kidney Int [Internet]. 2008 Oct 2 [cited 2024 Jul 15];74(8):998–1003. Available from: https://www.kidney-international.org/article/S0085-2538(15)53472-8/fulltext 10.1038/ki.2008.336 18633345

[127] Hadi Z, Ahmadi E, Shams-Esfandabadi N, Davoodian N, Shirazi A, Moradian M. Polyvinyl alcohol addition to freezing extender can improve the post-thaw quality, longevity and in vitro fertility of ram epididymal spermatozoa. Cryobiology [Internet]. 2024 Mar 1 [cited 2024 Jul 15];114:104853. Available from: https://www.sciencedirect.com/science/article/pii/S0011224024000087 10.1016/j.cryobiol.2024.104853 38301951

[128] Smillie Ja, Munro A c., Wood Gc, Mitchell R. Cryopreservation of human platelets with polyvinylpyrrolidone. Transfusion (Paris) [Internet]. 1981 [cited 2024 Jul 15];21(5):552–6. Available from: https://onlinelibrary.wiley.com/doi/abs/10.1046/j.1537-2995.1981.21582040818.x 10.1046/j.1537-2995.1981.21582040818.x 6170136

[129] Wang M, Mahajan A, Miller JS, McKenna DH, Aksan A. Physicochemical Mechanisms of Protection Offered by Agarose Encapsulation during Cryopreservation of Mammalian Cells in the Absence of Membrane Penetrating Cryoprotectants. ACS Appl Bio Mater [Internet]. 2023 Jun 19 [cited 2025 Apr 23];6(6):2226–36. Available from: https://www.ncbi.nlm.nih.gov/pmc/articles/PMC10330259/10.1021/acsabm.3c00098 PMC10330259 37212878

[130] Kurihara M, Fukushima,Mai M, Akane, Tanaka, Takashi, Sugimoto, Kouhei, and, Okada H. Comparative study of agarose-gel microcapsules and Cryotop in cryopreservation of extremely small numbers of human spermatozoa. Syst Biol Reprod Med [Internet]. 2021 May 4 [cited 2025 Apr 23];67(3):244–50. Available from: 10.1080/19396368.2021.1873457 10.1080/19396368.2021.1873457 33939593

[131] Wang X, Xu H. Incorporation of DMSO and dextran-40 into a gelatin/alginate hydrogel for controlled assembled cell cryopreservation. Cryobiology [Internet]. 2010 Dec 1 [cited 2025 Apr 23];61(3):345–51. Available from: https://www.sciencedirect.com/science/article/pii/S001122401000324X 10.1016/j.cryobiol.2010.10.161 21055398

[132] Rosner SR, Ram-Mohan S, Paez-Cortez JR, Lavoie TL, Dowell ML, Yuan L et al. Airway Contractility in the Precision-Cut Lung Slice after Cryopreservation. Am J Respir Cell Mol Biol [Internet]. 2014 May [cited 2024 Jul 1];50(5):876–81. Available from: https://www.ncbi.nlm.nih.gov/pmc/articles/PMC4068941/10.1165/rcmb.2013-0166MA PMC4068941 24313705

[133] Katsen-Globa A, Meiser I, Petrenko YA, Ivanov RV, Lozinsky VI, Zimmermann H et al. Towards ready-to-use 3-D scaffolds for regenerative medicine: adhesion-based cryopreservation of human mesenchymal stem cells attached and spread within alginate–gelatin cryogel scaffolds. J Mater Sci Mater Med [Internet]. 2014 [cited 2025 Apr 23];25(3):857–71. Available from: https://www.ncbi.nlm.nih.gov/pmc/articles/PMC3942626/10.1007/s10856-013-5108-x PMC3942626 24297514

[134] Ishizaki T, Tanaka D, Ishibashi K, Takahashi K, Hirata E, Kuroda K. Cell Damage Mechanisms during Cryopreservation in a Zwitterion Solution and Its Alleviation by DMSO. J Phys Chem B [Internet]. 2024 Apr 25 [cited 2024 Jun 28];128(16):3904–9. Available from: 10.1021/acs.jpcb.3c07773 10.1021/acs.jpcb.3c07773 38613503

[135] Rienzi L, Martinez F, Ubaldi F, Minasi MG, Iacobelli M, Tesarik J et al. Polscope analysis of meiotic spindle changes in living metaphase II human oocytes during the freezing and thawing procedures. Hum Reprod [Internet]. 2004 Mar 1 [cited 2024 Dec 3];19(3):655–9. Available from: 10.1093/humrep/deh101 10.1093/humrep/deh101 14998966

[136] Mitchell DE, Lilliman M, Spain SG, Gibson MI. Quantitative study on the antifreeze protein mimetic ice growth inhibition properties of poly(ampholytes) derived from vinyl-based polymers. Biomater Sci [Internet]. 2014 Oct 28 [cited 2024 Dec 3];2(12):1787–95. Available from: https://pubs.rsc.org/en/content/articlelanding/2014/bm/c4bm00153b 10.1039/c4bm00153b 32481956

[137] Biggs CI, Stubbs C, Graham B, Fayter AER, Hasan M, Gibson MI. Mimicking the Ice Recrystallization Activity of Biological Antifreezes. When is a New Polymer Active? Macromol Biosci [Internet]. 2019 [cited 2024 Dec 3];19(7):1900082. Available from: https://onlinelibrary.wiley.com/doi/abs/10.1002/mabi.201900082 10.1002/mabi.201900082 PMC6828557 31087781

[138] Matsumoto S, Matsusita M, Morita T, Kamachi H, Tsukiyama S, Furukawa Y et al. Effects of synthetic antifreeze glycoprotein analogue on islet cell survival and function during cryopreservation. Cryobiology [Internet]. 2006 Feb 1 [cited 2024 Jun 28];52(1):90–8. Available from: https://www.sciencedirect.com/science/article/pii/S0011224005001562 10.1016/j.cryobiol.2005.10.010 16325794

[139] Toxopeus J, Sinclair BJ. Mechanisms underlying insect freeze tolerance. Biol Rev [Internet]. 2018 [cited 2024 Dec 3];93(4):1891–914. Available from: https://onlinelibrary.wiley.com/doi/abs/10.1111/brv.12425 10.1111/brv.12425 29749114

[140] Costanzo JP, Lee RE. Cryoprotection by urea in a terrestrially hibernating frog. J Exp Biol [Internet]. 2005 Nov 1 [cited 2024 Sep 13];208(21):4079–89. Available from: 10.1242/jeb.01859 10.1242/jeb.01859 16244167

[141] Lovelock JE. The protective action of neutral solutes against haemolysis by freezing and thawing. Biochem J [Internet]. 1954 Feb 1 [cited 2024 Jun 26];56(2):265–70. Available from: 10.1042/bj0560265 10.1042/bj0560265 PMC1269611 13140185

[142] Lee CY, Bastacky J. Comparative Mathematical Analyses of Freezing in Lung and Solid Tissue. Cryobiology [Internet]. 1995 Aug 1 [cited 2024 Jun 25];32(4):299–305. Available from: https://www.sciencedirect.com/science/article/pii/S0011224085710292 10.1006/cryo.1995.1029 7656563

[143] Hara J, Tottori J, Anders M, Dadhwal S, Asuri P, Mobed-Miremadi M. Trehalose effectiveness as a cryoprotectant in 2D and 3D cell cultures of human embryonic kidney cells. 2017. 10.3109/21691401.2016.1167698 27050441

[144] Freeman BA, O’Neil JJ. Tissue slices in the study of lung metabolism and toxicology. Environ Health Perspect [Internet]. 1984 Jun [cited 2024 Jun 29];56:51–60. Available from: https://www.ncbi.nlm.nih.gov/pmc/articles/PMC1568205/10.1289/ehp.845651 PMC1568205 6383802

[145] Karl PI, Friedman PA. Competition between paraquat and putrescine for accumulation by rat lung slices. Toxicology [Internet]. 1983 Mar 1 [cited 2024 Jun 29];26(3):317–23. Available from: https://www.sciencedirect.com/science/article/pii/0300483X83900926 10.1016/0300-483x(83)90092-6 6857703

[146] Smith LL, Wyatt I, Rose MS. Factors affecting the efflux of paraquat from rat lung slices. Toxicology [Internet]. 1981 Jan 1 [cited 2024 Jun 29];19(3):197–207. Available from: https://www.sciencedirect.com/science/article/pii/0300483X81901293 10.1016/0300-483x(81)90129-3 7233444

[147] Fisher RL, Smith MS, Hasal SJ, Hasal KS, Gandolfi AJ, Brendel K. The Use of Human Lung Slices in Toxicology. Hum Exp Toxicol [Internet]. 1994 Jul 1 [cited 2024 Jun 29];13(7):466–71. Available from: 10.1177/096032719401300703 10.1177/096032719401300703 7917502

[148] Generation of Human 3D Lung Tissue Cultures 3D. -LTCs for Disease Modeling [Internet]. [cited 2024 Jun 29]. Available from: https://app.jove.com/t/58437 10.3791/58437 30829341

[149] Michalaki C, Dean C, Johansson C. The Use of Precision-Cut Lung Slices for Studying Innate Immunity to Viral Infections. Curr Protoc [Internet]. 2022 Aug [cited 2024 Jun 29];2(8):e505. Available from: https://www.ncbi.nlm.nih.gov/pmc/articles/PMC9545600/10.1002/cpz1.505 PMC9545600 35938685

[150] Crue T, Lee GY, Peng JY, chun, Schaunaman N, Agraval H, Day BJ et al. Single cell RNA-sequencing of human precision-cut lung slices: A novel approach to study the effect of vaping and viral infection on lung health. Innate Immun [Internet]. 2023 Jul [cited 2024 Jun 29];29(5):61–70. Available from: https://www.ncbi.nlm.nih.gov/pmc/articles/PMC10357887/10.1177/17534259231181029 PMC10357887 37306239

[151] Switalla S, Knebel J, Ritter D, Krug N, Braun A, Sewald K. Effects of acute *in vitro* exposure of murine precision-cut lung slices to gaseous nitrogen dioxide and ozone in an air–liquid interface (ALI) culture. Toxicol Lett [Internet]. 2010 Jul 1 [cited 2024 Jun 29];196(2):117–24. Available from: https://www.sciencedirect.com/science/article/pii/S0378427410013093 10.1016/j.toxlet.2010.04.004 20394810

[152] Schlepütz M, Uhlig S, Martin C. Electric field stimulation of precision-cut lung slices. J Appl Physiol [Internet]. 2011 Feb [cited 2025 Apr 23];110(2):545–54. Available from: https://journals.physiology.org/doi/full/10.1152/japplphysiol.00409.2010 10.1152/japplphysiol.00409.2010 21109600

[153] Koziol-White CJ, Yoo EJ, Cao G, Zhang J, Papanikolaou E, Pushkarsky I et al. Inhibition of PI3K promotes dilation of human small airways in a rho kinase‐dependent manner. Br J Pharmacol [Internet]. 2016 Sep [cited 2024 Jun 29];173(18):2726–38. Available from: https://www.ncbi.nlm.nih.gov/pmc/articles/PMC4995285/10.1111/bph.13542 PMC4995285 27352269

[154] Hempel P, Klein V, Michely A, Böll S, Rieg AD, Spillner J et al. Amitriptyline inhibits bronchoconstriction and directly promotes dilatation of the airways. Respir Res [Internet]. 2023 [cited 2024 Jun 29];24:262. Available from: https://www.ncbi.nlm.nih.gov/pmc/articles/PMC10617234/10.1186/s12931-023-02580-6 PMC10617234 37907918

[155] Lavoie TL, Krishnan R, Siegel HR, Maston ED, Fredberg JJ, Solway J et al. Dilatation of the Constricted Human Airway by Tidal Expansion of Lung Parenchyma. Am J Respir Crit Care Med [Internet]. 2012 Aug 1 [cited 2025 Apr 23];186(3):225–32. Available from: https://www.ncbi.nlm.nih.gov/pmc/articles/PMC3423451/10.1164/rccm.201202-0368OC PMC3423451 22679010

[156] Kim JH, Schaible N, Hall JK, Bartolák-Suki E, Deng Y, Herrmann J et al. Multiscale stiffness of human emphysematous precision cut lung slices. Sci Adv [Internet]. [cited 2025 Apr 23];9(20):eadf2535. Available from: https://www.ncbi.nlm.nih.gov/pmc/articles/PMC10198632/10.1126/sciadv.adf2535 PMC10198632 37205750

[157] Watson CY, Damiani F, Ram-Mohan S, Rodrigues S, de Moura Queiroz P, Donaghey TC et al. Screening for Chemical Toxicity Using Cryopreserved Precision Cut Lung Slices. Toxicol Sci [Internet]. 2016 Mar [cited 2024 Jun 30];150(1):225–33. Available from: https://www.ncbi.nlm.nih.gov/pmc/articles/PMC5009619/10.1093/toxsci/kfv320 PMC5009619 26719368

[158] Cooper PR, Zhang J, Damera G, Hoshi T, Zopf DA, Panettieri RA. C-027 Inhibits IgE-mediated passive sensitization bronchoconstriction and acts as a histamine and serotonin antagonist in human airways. Allergy Asthma Proc Off J Reg State Allergy Soc [Internet]. 2011 [cited 2024 Jun 30];32(5):359–65. Available from: https://www.ncbi.nlm.nih.gov/pmc/articles/PMC3968313/10.2500/aap.2011.32.3460 PMC3968313 22195688

[159] Blomberg R, Sompel K, Hauer C, Smith AJ, Peña B, Driscoll J et al. Hydrogel-Embedded Precision-Cut Lung Slices Model Lung Cancer Premalignancy Ex Vivo. Adv Healthc Mater [Internet]. 2024 [cited 2024 Jun 30];13(4):2302246. Available from: https://onlinelibrary.wiley.com/doi/abs/10.1002/adhm.202302246 10.1002/adhm.202302246 PMC10872976 37953708

[160] Lee JY, Reyes NS, Ravishankar S, Zhou M, Krasilnikov M, Ringler C et al. An in vivo screening platform identifies senolytic compounds that target p16INK4a + fibroblasts in lung fibrosis. J Clin Invest [Internet]. [cited 2024 Jun 30];134(9):e173371. Available from: https://www.ncbi.nlm.nih.gov/pmc/articles/PMC11060735/10.1172/JCI173371 PMC11060735 38451724

[161] Lehmann M, Buhl L, Alsafadi HN, Klee S, Hermann S, Mutze K et al. Differential effects of Nintedanib and Pirfenidone on lung alveolar epithelial cell function in ex vivo murine and human lung tissue cultures of pulmonary fibrosis. Respir Res [Internet]. 2018 [cited 2024 Jun 30];19:175. Available from: https://www.ncbi.nlm.nih.gov/pmc/articles/PMC6138909/10.1186/s12931-018-0876-y PMC6138909 30219058

[162] Bailey KE, Pino C, Lennon ML, Lyons A, Jacot JG, Lammers SR et al. Embedding of Precision-Cut Lung Slices in Engineered Hydrogel Biomaterials Supports Extended Ex Vivo Culture. Am J Respir Cell Mol Biol [Internet]. 2020 Jan [cited 2024 Jul 1];62(1):14–22. Available from: https://www.ncbi.nlm.nih.gov/pmc/articles/PMC6938134/10.1165/rcmb.2019-0232MA PMC6938134 31513744

[163] Tigges J, Eggerbauer F, Worek F, Thiermann H, Rauen U, Wille T. Optimization of long-term cold storage of rat precision-cut lung slices with a tissue preservation solution. Am J Physiol-Lung Cell Mol Physiol [Internet]. 2021 Dec [cited 2024 Jun 25];321(6):L1023–35. Available from: 10.1152/ajplung.00076.2021 10.1152/ajplung.00076.2021 34643087

[164] Bull DA, Connors RC, Reid BB, Albanil A, Stringham JC, Karwande SV. Improved Biochemical Preservation of Lung Slices during Cold Storage. J Surg Res [Internet]. 2000 May 15 [cited 2024 Jul 1];90(2):144–8. Available from: https://www.journalofsurgicalresearch.com/article/S0022-4804(00)95870-0/abstract 10.1006/jsre.2000.5870 10792955

[165] Pechous RD, Malaviarachchi PA, Banerjee SK, Byrum SD, Alkam DH, Ghaffarieh A et al. An ex vivo human precision-cut lung slice platform provides insight into SARS-CoV-2 pathogenesis and antiviral drug efficacy. J Virol [Internet]. 2024 Jun 28 [cited 2024 Jul 1];0(0):e00794-24. Available from: 10.1128/jvi.00794-24 10.1128/jvi.00794-24 PMC11265413 38940558

[166] Panoskaltsis-Mortari A. Bioreactor Development for Lung Tissue Engineering. Curr Transplant Rep [Internet]. 2015 Mar [cited 2024 Jul 1];2(1):90–7. Available from: https://www.ncbi.nlm.nih.gov/pmc/articles/PMC4339073/10.1007/s40472-014-0048-z PMC4339073 25729638

[167] Moreno Garijo J, Roscoe A. Ex-vivo lung perfusion. Curr Opin Anesthesiol [Internet]. 2020 Feb [cited 2024 Jul 1];33(1):50. Available from: https://journals.lww.com/co-anesthesiology/abstract/2020/02000/ex_vivo_lung_perfusion.8.aspx 10.1097/ACO.0000000000000804 31688085

[168] Köppen K, Fatykhova D, Holland G, Rauch J, Tappe D, Graff M et al. Ex vivo infection model for Francisella using human lung tissue. Front Cell Infect Microbiol [Internet]. 2023 Jul 10 [cited 2024 Jun 24];13:1224356. Available from: https://www.ncbi.nlm.nih.gov/pmc/articles/PMC10365108/10.3389/fcimb.2023.1224356 PMC10365108 37492528

[169] Szymanski KV, Toennies M, Becher A, Fatykhova D, N’Guessan PD, Gutbier B et al. Streptococcus pneumoniae-induced regulation of cyclooxygenase-2 in human lung tissue. Eur Respir J [Internet]. 2012 Dec 1 [cited 2024 Jul 1];40(6):1458–67. Available from: https://erj.ersjournals.com/content/40/6/1458 10.1183/09031936.00186911 22441740

[170] Hönzke K, Obermayer B, Mache C, Fathykova D, Kessler M, Dökel S et al. Human lungs show limited permissiveness for SARS-CoV-2 due to scarce ACE2 levels but virus-induced expansion of inflammatory macrophages. Eur Respir J [Internet]. 2022 Dec 1 [cited 2024 Jul 1];60(6):2102725. Available from: https://www.ncbi.nlm.nih.gov/pmc/articles/PMC9712848/10.1183/13993003.02725-2021 PMC9712848 35728978

[171] Hocke AC, Becher A, Knepper J, Peter A, Holland G, Tönnies M et al. Emerging Human Middle East Respiratory Syndrome Coronavirus Causes Widespread Infection and Alveolar Damage in Human Lungs. Am J Respir Crit Care Med [Internet]. 2013 Oct [cited 2024 Jul 1];188(7):882–6. Available from: https://www.atsjournals.org/doi/10.1164/rccm.201305-0954LE 10.1164/rccm.201305-0954LE 24083868

[172] Jäger J, Marwitz S, Tiefenau J, Rasch J, Shevchuk O, Kugler C et al. Human Lung Tissue Explants Reveal Novel Interactions during Legionella pneumophila Infections. Infect Immun [Internet]. 2014 Jan [cited 2024 Jul 1];82(1):275–85. Available from: https://www.ncbi.nlm.nih.gov/pmc/articles/PMC3911869/10.1128/IAI.00703-13 PMC3911869 24166955

[173] Peter A, Fatykhova D, Kershaw O, Gruber AD, Rueckert J, Neudecker J et al. Localization and pneumococcal alteration of junction proteins in the human alveolar–capillary compartment. Histochem Cell Biol [Internet]. 2017 Jun 1 [cited 2024 Jul 1];147(6):707–19. Available from: 10.1007/s00418-017-1551-y 10.1007/s00418-017-1551-y 28247028

[174] Diodati N, Dupee Z, Lima F, Famiglietti J, Smolchek R, Qu G et al. 3D Culture Analysis of Cancer Cell Adherence to Ex Vivo Lung Microexplants. Tissue Eng Part C Methods [Internet]. 2024 Jul 30 [cited 2024 Aug 8]; Available from: https://www.liebertpub.com/doi/10.1089/ten.TEC.2024.0146 10.1089/ten.tec.2024.0146 PMC12394800 39078332

[175] He A, Powell S, Kyle M, Rose M, Masmila E, Estrada V et al. Cryopreservation of Viable Human Tissues: Renewable Resource for Viable Tissue, Cell Lines, and Organoid Development. Biopreservation Biobanking [Internet]. 2020 Jun 1 [cited 2024 Jul 7];18(3):222–7. Available from: https://www.ncbi.nlm.nih.gov/pmc/articles/PMC7310214/10.1089/bio.2019.0062 PMC7310214 32302515

[176] Nguyen DT, Famiglietti JE, Smolchek RA, Dupee Z, Diodati N, Pedro DI, et al. 3D in vitro platform for cell and explant culture in Liquid-like solids. Cells. 2022;11(6):967. 35326418 10.3390/cells11060967 PMC8946834

[177] Nicholas B, Staples KJ, Moese S, Meldrum E, Ward J, Dennison P et al. A Novel Lung Explant Model for the Ex Vivo Study of Efficacy and Mechanisms of Anti-Influenza Drugs. J Immunol Author Choice [Internet]. 2015 Jun 15 [cited 2024 Jul 1];194(12):6144–54. Available from: https://www.ncbi.nlm.nih.gov/pmc/articles/PMC4456633/10.4049/jimmunol.1402283 PMC4456633 25934861

[178] Nguyen DT, Pedro DI, Pepe A, Rosa JG, Bowman JI, Trachsel L, et al. Bioconjugation of COL1 protein on liquid-like solid surfaces to study tumor invasion dynamics. Biointerphases. 2023;18(2):021001. 36898958 10.1116/6.0002083 PMC10008099

[179] Lautner LJ, Freed DH, Nagendran J, Acker JP. Current techniques and the future of lung preservation. Cryobiology [Internet]. 2020 Jun 1 [cited 2024 Jun 25];94:1–8. Available from: https://www.sciencedirect.com/science/article/pii/S0011224020301358 10.1016/j.cryobiol.2020.04.009 32361000

[180] Arutyunyan I, Elchaninov A, Sukhikh G, Fatkhudinov T. Cryopreservation of Tissue-Engineered Scaffold-Based Constructs: from Concept to Reality. Stem Cell Rev Rep [Internet]. 2022 Apr 1 [cited 2024 Dec 9];18(4):1234–52. Available from: 10.1007/s12015-021-10299-4 10.1007/s12015-021-10299-4 34761366

[181] Stabler CT, Lecht S, Mondrinos MJ, Goulart E, Lazarovici P, Lelkes PI. Revascularization of decellularized lung scaffolds: principles and progress. Am J Physiol-Lung Cell Mol Physiol [Internet]. 2015 Dec 1 [cited 2024 Jun 29];309(11):L1273–85. Available from: https://www.physiology.org/doi/10.1152/ajplung.00237.2015 10.1152/ajplung.00237.2015 PMC4669341 26408553

[182] Calle EA, Hill RC, Leiby KL, Le AV, Gard AL, Madri JA et al. Targeted proteomics effectively quantifies differences between native lung and detergent-decellularized lung extracellular matrices. Acta Biomater [Internet]. 2016 Dec [cited 2024 Jun 29];46:91–100. Available from: https://www.ncbi.nlm.nih.gov/pmc/articles/PMC5451113/10.1016/j.actbio.2016.09.043 PMC5451113 27693690

[183] Uriarte JJ, Uhl FE, Rolandsson Enes SE, Pouliot RA, Weiss DJ. Lung bioengineering: advances and challenges in lung decellularization and recellularization. Curr Opin Organ Transplant [Internet]. 2018 Dec [cited 2024 Jun 29];23(6):673–8. Available from: https://www.ncbi.nlm.nih.gov/pmc/articles/PMC8669574/10.1097/MOT.0000000000000584 PMC8669574 30300330

[184] Ghaedi M, Le AV, Hatachi G, Beloiartsev A, Rocco K, Sivarapatna A et al. Bioengineered lungs generated from human iPSCs-derived epithelial cells on native extracellular matrix. J Tissue Eng Regen Med [Internet]. 2018 Mar [cited 2024 Jun 29];12(3):e1623–35. Available from: https://www.ncbi.nlm.nih.gov/pmc/articles/PMC5991621/10.1002/term.2589 PMC5991621 29024475

[185] Rosmark O, Åhrman E, Müller C, Elowsson Rendin L, Eriksson L, Malmström A et al. Quantifying extracellular matrix turnover in human lung scaffold cultures. Sci Rep [Internet]. 2018 Apr 3 [cited 2024 Jul 7];8:5409. Available from: https://www.ncbi.nlm.nih.gov/pmc/articles/PMC5882971/10.1038/s41598-018-23702-x PMC5882971 29615673

[186] Kim J, Koo BK, Knoblich JA. Human organoids: model systems for human biology and medicine. Nat Rev Mol Cell Biol [Internet]. 2020 [cited 2024 Jul 9];21(10):571–84. Available from: https://www.ncbi.nlm.nih.gov/pmc/articles/PMC7339799/10.1038/s41580-020-0259-3 PMC7339799 32636524

[187] Naba A, Clauser KR, Hoersch S, Liu H, Carr SA, Hynes RO. The Matrisome: In Silico Definition and In Vivo Characterization by Proteomics of Normal and Tumor Extracellular Matrices. Mol Cell Proteomics MCP [Internet]. 2012 Apr [cited 2024 Jul 7];11(4):M111.014647. Available from: https://www.ncbi.nlm.nih.gov/pmc/articles/PMC3322572/10.1074/mcp.M111.014647 PMC3322572 22159717

[188] Balestrini JL, Gard AL, Gerhold KA, Wilcox EC, Liu A, Schwan J et al. Comparative Biology of Decellularized Lung Matrix: Implications of Species Mismatch in Regenerative Medicine. Biomaterials [Internet]. 2016 Sep [cited 2024 Jul 8];102:220–30. Available from: https://www.ncbi.nlm.nih.gov/pmc/articles/PMC4939101/10.1016/j.biomaterials.2016.06.025 PMC4939101 27344365

[189] Gilpin SE, Guyette JP, Gonzalez G, Ren X, Asara JM, Mathisen DJ et al. Perfusion decellularization of human and porcine lungs: Bringing the matrix to clinical scale. J Heart Lung Transplant [Internet]. 2014 Mar 1 [cited 2024 Jul 7];33(3):298–308. Available from: https://www.jhltonline.org/article/S1053-2498(13)01511-8/abstract 10.1016/j.healun.2013.10.030 24365767

[190] Murray LA. Commonalities between the pro-fibrotic mechanisms in COPD and IPF. Pulm Pharmacol Ther [Internet]. 2012 Aug 1 [cited 2024 Jul 7];25(4):276–80. Available from: https://www.sciencedirect.com/science/article/pii/S1094553911001416 10.1016/j.pupt.2011.08.003 21983244

[191] Postma DS, Timens W. Remodeling in Asthma and Chronic Obstructive Pulmonary Disease. Proc Am Thorac Soc [Internet]. 2006 Jul [cited 2024 Jul 7];3(5):434–9. Available from: https://www.atsjournals.org/doi/full/10.1513/pats.200601-006AW 10.1513/pats.200601-006AW 16799088

[192] Mahfouzi SH, Safiabadi Tali SH, Amoabediny G. Decellularized Human-Sized Pulmonary Scaffolds for Lung Tissue Engineering: A Comprehensive Review. Regen Med [Internet]. 2021 Aug 1 [cited 2024 Jul 8];16(8):757–74. Available from: 10.2217/rme-2020-0152 10.2217/rme-2020-0152 34431331

[193] Taka S, Nikopoulou C, Polyzos A, Megremis S, Skevaki CL, Roumpedaki E et al. Effects of cryopreservation on antiviral responses of primary airway epithelial cells. Allergy [Internet]. 2020 [cited 2024 Jul 7];75(6):1486–9. Available from: https://onlinelibrary.wiley.com/doi/abs/10.1111/all.14163 10.1111/all.14163 31879965

[194] Bibevski S, Ruzmetov M, Fortuna RS, Turrentine MW, Brown JW, Ohye RG. Performance of SynerGraft Decellularized Pulmonary Allografts Compared With Standard Cryopreserved Allografts: Results From Multiinstitutional Data. Ann Thorac Surg [Internet]. 2017 Mar 1 [cited 2024 Dec 9];103(3):869–74. Available from: https://www.annalsthoracicsurgery.org/article/S0003-4975(16)30977-8/fulltext 10.1016/j.athoracsur.2016.07.068 27788940

[195] Smith E, Cochrane WJ. CYSTIC ORGANOID TERATOMA. Can Med Assoc J [Internet]. 1946 Aug [cited 2024 Jul 9];55(2):151–2. Available from: https://www.ncbi.nlm.nih.gov/pmc/articles/PMC1582935/20992760

[196] Cruz-Acuña R, Quirós M, Farkas AE, Dedhia PH, Huang S, Siuda D et al. Synthetic Hydrogels for Human Intestinal Organoid Generation and Colonic Wound Repair. Nat Cell Biol [Internet]. 2017 Nov [cited 2024 Jul 9];19(11):1326–35. Available from: https://www.ncbi.nlm.nih.gov/pmc/articles/PMC5664213/10.1038/ncb3632 PMC5664213 29058719

[197] Hofer M, Lutolf MP. Engineering organoids. Nat Rev Mater [Internet]. 2021 [cited 2024 Jul 9];6(5):402–20. Available from: https://www.ncbi.nlm.nih.gov/pmc/articles/PMC7893133/10.1038/s41578-021-00279-y PMC7893133 33623712

[198] Mondrinos MJ, Koutzaki S, Jiwanmall E, Li M, Dechadarevian JP, Lelkes PI et al. Engineering Three-Dimensional Pulmonary Tissue Constructs. Tissue Eng [Internet]. 2006 Apr [cited 2024 Jul 9];12(4):717–28. Available from: https://www.liebertpub.com/doi/10.1089/ten.2006.12.717 10.1089/ten.2006.12.717 16674286

[199] Barkauskas CE, Cronce MJ, Rackley CR, Bowie EJ, Keene DR, Stripp BR et al. Type 2 alveolar cells are stem cells in adult lung. J Clin Invest [Internet]. 2013 Jul 1 [cited 2024 Jul 9];123(7):3025–36. Available from: https://www.ncbi.nlm.nih.gov/pmc/articles/PMC3696553/10.1172/JCI68782 PMC3696553 23921127

[200] Dye BR, Hill DR, Ferguson MA, Tsai YH, Nagy MS, Dyal R et al. In vitro generation of human pluripotent stem cell derived lung organoids. eLife [Internet]. [cited 2024 Jul 9];4:e05098. Available from: https://www.ncbi.nlm.nih.gov/pmc/articles/PMC4370217/10.7554/eLife.05098 PMC4370217 25803487

[201] Vilà-González M, Pinte L, Fradique R, Causa E, Kool H, Rodrat M et al. In vitro platform to model the function of ionocytes in the human airway epithelium. Respir Res [Internet]. 2024 [cited 2024 Jul 9];25:180. Available from: https://www.ncbi.nlm.nih.gov/pmc/articles/PMC11045446/10.1186/s12931-024-02800-7 PMC11045446 38664797

[202] Frankowski J, Kurzątkowska M, Sobczak M, Piotrowska U. Utilization of 3D bioprinting technology in creating human tissue and organoid models for preclinical drug research– State-of-the-art. Int J Pharm [Internet]. 2023 Sep 25 [cited 2024 Jul 9];644:123313. Available from: https://www.sciencedirect.com/science/article/pii/S0378517323007330 10.1016/j.ijpharm.2023.123313 37579828

[203] Gerli MFM, Calà G, Beesley MA, Sina B, Tullie L, Sun KY et al. Single-cell guided prenatal derivation of primary fetal epithelial organoids from human amniotic and tracheal fluids. Nat Med [Internet]. 2024 [cited 2024 Jul 9];30(3):875–87. Available from: https://www.ncbi.nlm.nih.gov/pmc/articles/PMC10957479/10.1038/s41591-024-02807-z PMC10957479 38438734

[204] Gentemann L, Donath S, Seidler AE, Patyk L, Buettner M, Heisterkamp A et al. Mimicking acute airway tissue damage using femtosecond laser nanosurgery in airway organoids. Front Cell Dev Biol [Internet]. 2023 Sep 8 [cited 2024 Jul 9];11:1268621. Available from: https://www.ncbi.nlm.nih.gov/pmc/articles/PMC10514509/10.3389/fcell.2023.1268621 PMC10514509 37745302

[205] Mondrinos MJ, Jones PL, Finck CM, Lelkes PI. Engineering De Novo Assembly of Fetal Pulmonary Organoids. Tissue Eng Part A [Internet]. 2014 Nov 1 [cited 2024 Jul 9];20(21–22):2892–907. Available from: https://www.ncbi.nlm.nih.gov/pmc/articles/PMC4229698/10.1089/ten.tea.2014.0085 PMC4229698 24825442

[206] Choi Ymi, Lee H, Ann M, Song M, Rheey J, Jang J. 3D bioprinted vascularized lung cancer organoid models with underlying disease capable of more precise drug evaluation. Biofabrication [Internet]. 2023 Jun [cited 2024 Jul 9];15(3):034104. Available from: 10.1088/1758-5090/acd95f 10.1088/1758-5090/acd95f 37236168

[207] Lee SY, Cho HJ, Choi J, Ku B, Moon SW, Moon MH et al. Cancer organoid-based diagnosis reactivity prediction (CODRP) index-based anticancer drug sensitivity test in ALK-rearrangement positive non-small cell lung cancer (NSCLC). J Exp Clin Cancer Res CR [Internet]. 2023 Nov 22 [cited 2024 Jul 9];42:309. Available from: https://www.ncbi.nlm.nih.gov/pmc/articles/PMC10664561/10.1186/s13046-023-02899-4 PMC10664561 37993887

[208] Zhang XS, Xie G, Ma H, Ding S, Wu YX, Fei Y et al. Highly reproducible and cost-effective one-pot organoid differentiation using a novel platform based on PF-127 triggered spheroid assembly. Biofabrication [Internet]. 2023 Aug [cited 2024 Jul 9];15(4):045014. Available from: 10.1088/1758-5090/acee21 10.1088/1758-5090/acee21 37552975

[209] Hynds RE, Butler CR, Janes SM, Giangreco A. Expansion of Human Airway Basal Stem Cells and Their Differentiation as 3D Tracheospheres. In: Turksen K, editor. Organoids: Stem Cells, Structure, and Function [Internet]. New York, NY: Springer; 2019 [cited 2024 Jul 10]. pp. 43–53. Available from: 10.1007/7651_2016_5 10.1007/7651_2016_5 27539459

[210] Hui KPY, Ching RHH, Chan SKH, Nicholls JM, Sachs N, Clevers H et al. Tropism, replication competence, and innate immune responses of influenza virus: an analysis of human airway organoids and ex-vivo bronchus cultures. Lancet Respir Med [Internet]. 2018 Nov 1 [cited 2024 Jul 9];6(11):846–54. Available from: https://www.thelancet.com/journals/lanres/article/PIIS2213-2600(18)30236-4/abstract 10.1016/S2213-2600(18)30236-4 30001996

[211] Lim K, Donovan APA, Tang W, Sun D, He P, Pett JP et al. Organoid modeling of human fetal lung alveolar development reveals mechanisms of cell fate patterning and neonatal respiratory disease. Cell Stem Cell [Internet]. 2023 Jan 5 [cited 2024 Jul 9];30(1):20–37.e9. Available from: https://www.cell.com/cell-stem-cell/abstract/S1934-5909(22)00460-X 10.1016/j.stem.2022.11.013 PMC7618456 36493780

[212] Nikolić MZ, Caritg O, Jeng Q, Johnson JA, Sun D, Howell KJ et al. Human embryonic lung epithelial tips are multipotent progenitors that can be expanded in vitro as long-term self-renewing organoids. eLife [Internet]. [cited 2024 Jul 9];6:e26575. Available from: https://www.ncbi.nlm.nih.gov/pmc/articles/PMC5555721/10.7554/eLife.26575 PMC5555721 28665271

[213] Dey MK, Devireddy RV. Adult Stem Cells Freezing Processes and Cryopreservation Protocols. In: Gimble J, Bunnell B, Frazier T, Sanchez C, editors. Adipose-Derived Stem Cells: Methods and Protocols [Internet]. New York, NY: Springer US; 2024 [cited 2024 Jul 10]. pp. 53–89. Available from: 10.1007/978-1-0716-3762-3_5 10.1007/978-1-0716-3762-3_5 38478226

[214] Rogulska O, Havelkova J, Petrenko Y. Cryopreservation of Organoids. Cryoletters [Internet]. 2023 Mar 1 [cited 2024 Jul 10];44(2):65–75. Available from: https://www.ingentaconnect.com/content/10.54680/fr23210110112 37883156

[215] Thege FI, Rupani DN, Barathi BB, Manning SL, Maitra A, Rhim AD et al. A programmable in vivo CRISPR activation model elucidates the oncogenic and immunosuppressive functions of MYC in lung adenocarcinoma. Cancer Res [Internet]. 2022 Aug 3 [cited 2024 Jul 10];82(15):2761–76. Available from: https://www.ncbi.nlm.nih.gov/pmc/articles/PMC9357118/10.1158/0008-5472.CAN-21-4009 PMC9357118 35666804

[216] Sun D, Evans L, Perrone F, Sokleva V, Lim K, Rezakhani S et al. A functional genetic toolbox for human tissue-derived organoids. eLife [Internet]. [cited 2024 Jul 10];10:e67886. Available from: https://www.ncbi.nlm.nih.gov/pmc/articles/PMC8553336/10.7554/eLife.67886 PMC8553336 34612202

[217] Kathiriya JJ, Wang C, Zhou M, Brumwell A, Cassandras M, Le Saux C et al. Human alveolar Type 2 epithelium transdifferentiates into metaplastic KRT5 + basal cells. Nat Cell Biol [Internet]. 2022 Jan [cited 2024 Jul 10];24(1):10–23. Available from: https://www.ncbi.nlm.nih.gov/pmc/articles/PMC8761168/10.1038/s41556-021-00809-4 PMC8761168 34969962

[218] Sato T, Stange DE, Ferrante M, Vries RGJ, van Es JH, Brink S, van den et al. Long-term Expansion of Epithelial Organoids From Human Colon, Adenoma, Adenocarcinoma, and Barrett’s Epithelium. Gastroenterology [Internet]. 2011 Nov 1 [cited 2024 Jul 10];141(5):1762–72. Available from: https://www.gastrojournal.org/article/S0016-5085(11)01108-5/fulltext?referrer=https%3A%2F%2Fpubmed.ncbi.nlm.nih.gov%2F 10.1053/j.gastro.2011.07.050 21889923

[219] Vaishnavi A, Juan J, Jacob M, Stehn C, Gardner EE, Scherzer MT et al. Transposon Mutagenesis Reveals RBMS3 Silencing as a Promoter of Malignant Progression of BRAFV600E-Driven Lung Tumorigenesis. Cancer Res [Internet]. 2022 Nov 15 [cited 2024 Jul 10];82(22):4261–73. Available from: https://www.ncbi.nlm.nih.gov/pmc/articles/PMC9664136/10.1158/0008-5472.CAN-21-3214 PMC9664136 36112789

[220] Studer L, Vera E, Cornacchia D, PROGRAMMING AND REPROGRAMMING CELLULAR AGE IN, THE ERA OF INDUCED PLURIPOTENCY. Cell Stem Cell [Internet]. 2015 Jun 4 [cited 2024 Jul 10];16(6):591–600. Available from: https://www.ncbi.nlm.nih.gov/pmc/articles/PMC4508309/10.1016/j.stem.2015.05.004 PMC4508309 26046759

[221] Hawkins FJ, Suzuki S, Beermann ML, Barillà C, Wang R, Villacorta-Martin C et al. Derivation of Airway Basal Stem Cells from Human Pluripotent Stem Cells. Cell Stem Cell [Internet]. 2021 Jan 7 [cited 2024 Jul 10];28(1):79–95.e8. Available from: https://www.ncbi.nlm.nih.gov/pmc/articles/PMC7796997/10.1016/j.stem.2020.09.017 PMC7796997 33098807

[222] Kim B, Lee E. Cryopreservation of engineered tissues and organoids. Organoid [Internet]. 2023 Dec 25 [cited 2024 Jul 10];3. Available from: http://j-organoid.org/journal/view.php?doi=10.51335/organoid.2023.3.e15

[223] Geraghty RJ, Capes-Davis A, Davis JM, Downward J, Freshney RI, Knezevic I et al. Guidelines for the use of cell lines in biomedical research. Br J Cancer [Internet]. 2014 Sep [cited 2024 Nov 20];111(6):1021–46. Available from: https://www.nature.com/articles/bjc2014166 10.1038/bjc.2014.166 PMC4453835 25117809

[224] Box GEP, Science, Statistics. J Am Stat Assoc [Internet]. 1976 [cited 2023 Aug 10];71(356):791–9. Available from: https://www.jstor.org/stable/2286841

